# The transcriptomic and proteomic responses of *Daphnia pulex* to changes in temperature and food supply comprise environment-specific and clone-specific elements

**DOI:** 10.1186/s12864-018-4742-6

**Published:** 2018-05-21

**Authors:** Dörthe Becker, Yann Reydelet, Jacqueline A. Lopez, Craig Jackson, John K. Colbourne, Susan Hawat, Michael Hippler, Bettina Zeis, Rüdiger J. Paul

**Affiliations:** 1 Institute of Zoophysiology, University of Münster, 48143 Münster, Germany; 2 Present address: Department of Biology, University of Virginia, Charlottesville, VA USA; 3 Present address: Genomics Core Facility, Galvin Life Science Center, University of Notre Dame, Notre Dame, IN USA; 4 Present address: School of Public and Environmental Affairs, Indiana University, Bloomington, IN USA; 5 Present address: Environmental Genomics Group, School of Biosciences, University of Birmingham, Edgbaston, Birmingham B15 2TT UK; 6 Institute of Plant Biology and Biotechnology, University of Münster, 48143 Münster, Germany

**Keywords:** Collagen, Heat shock protein, Heat stress, Hydrolase, Protein biosynthesis, Starvation, Stress protein, Temperature acclimation, Vitellogenin

## Abstract

**Background:**

Regulatory adjustments to acute and chronic temperature changes are highly important for aquatic ectotherms because temperature affects their metabolic rate as well as the already low oxygen concentration in water, which can upset their energy balance. This also applies to severe changes in food supply. Thus, we studied on a molecular level (transcriptomics and/or proteomics) the immediate responses to heat stress and starvation and the acclimation to different temperatures in two clonal isolates of the model microcrustacean *Daphnia pulex* from more or less stressful environments, which showed a higher (clone M) or lower (clone G) tolerance to heat and starvation.

**Results:**

The transcriptomic responses of clone G to acute heat stress (from 20 °C to 30 °C) and temperature acclimation (10 °C, 20 °C, and 24 °C) and the proteomic responses of both clones to acute heat, starvation, and heat-and-starvation stress comprised environment-specific and clone-specific elements. Acute stress (in particular heat stress) led to an early upregulation of stress genes and proteins (e.g., molecular chaperones) and a downregulation of metabolic genes and proteins (e.g., hydrolases). The transcriptomic responses to temperature acclimation differed clearly. They also varied depending on the temperature level. Acclimation to higher temperatures comprised an upregulation of metabolic genes and, in case of 24 °C acclimation, a downregulation of genes for translational processes and collagens. The proteomic responses of the clones M and G differed at any type of stress. Clone M showed markedly stronger and less stress-specific proteomic responses than clone G, which included the consistent expression of a specific heat shock protein (HSP60) and vitellogenin (VTG-SOD).

**Conclusions:**

The expression changes under acute stress can be interpreted as a switch from standard products of gene expression to stress-specific products. The expression changes under temperature acclimation probably served for an increase in energy intake (via digestion) and, if necessary, a decrease in energy expenditures (e.g, for translational processes). The stronger and less stress-specific proteomic responses of clone M indicate a lower degree of cell damage and an active preservation of the energy balance, which allowed adequate proteomic responses under stress, including the initiation of resting egg production (VTG-SOD expression) as an emergency reaction.

**Electronic supplementary material:**

The online version of this article (10.1186/s12864-018-4742-6) contains supplementary material, which is available to authorized users.

## Background

The microcrustacean *Daphnia pulex*, which inhabits thermally volatile ponds or small lakes, experiences severe acute and chronic temperature changes during diurnal vertical migration (minimal 10 °C) [1] and during the seasons (up to 20 °C) [2]. Concurrently, they face large fluctuations in nutrient (phytoplankton) availability [3]. As an immediate molecular response to acute temperature stress, these animals must counteract macromolecular damage [4] and temperature-induced ROS formation due to tissue hypoxia [5] by activating or expressing, for instance, molecular chaperones or antioxidative enzymes. Tissue hypoxia may result from a mismatch between oxygen supply and demand at more severe temperature changes (oxygen-and-capacity-limited temperature tolerance, OCLTT) [6]. Therefore, the low oxygen content of water, which further decreases with rising temperature and/or eutrophication, represents a particular risk for these ectothermic water breathers, particularly because their metabolic rate as well as their oxygen and energy demands additionally increase with rising temperature. Thus, adverse effects on their energy balance may occur, just as they may arise from a lowered nutrient availability and reduced energy supply. Consequently, both heat and starvation stress as extreme cases require parallel adjustments to improve the energy supply and/or to reduce the energy expenditures. Temperature acclimation as long-term adjustment to chronic temperature changes has been shown to involve, for instance, changes in membrane structure and enzyme composition [4]. In more general terms, the cellular or molecular responses to severe acute or chronic environmental changes have been classified as cellular stress response (CSR) or cellular homeostasis response (CHR) [7]. The CSR is the immediate and stressor-unspecific response found in all organisms from prokaryotes to man, which serves as a first line of defense against any kind of stress. One of its key functions is the provision of enough time for the later stressor-specific or even organism-specific CHR, which serves to recover cellular homeostasis.

Besides differences in the quantity and temporal course of environmental factors, the coexistence of genetically different clonal lineages of *Daphnia* in their natural habitats [8], which arises from the alteration between sexual and asexual reproduction (cyclical parthenogenesis), increases the variation range of animal-environment interactions. Several studies have already reported on selection by the spatiotemporal heterogeneity of the environment including effects of temperature or food on the genetic structure of *Daphnia* populations [9, 10]. Thus, the molecular responses of *Daphnia* to environmental changes may comprise clone-specific elements.

Previous transcriptomic studies on the effects of high temperature revealed a differential expression of entire gene groups in water breathers, including genes for molecular chaperones and proteolytic processes (genus *Mytilus*) [11] or for heat shock proteins, histones, apoptosis inhibitors, and enzymes involved in protein turnover (*Crassostrea gigas*) [12]. The air-breathing *Drosophila melanogaster* showed early upregulation of stress genes (e.g., for molecular chaperones and glutathione transferases) and early downregulation and/or a later upregulation of metabolic genes under heat stress [13]. Mammalian cells respond to heat stress through a highly coordinated upregulation of genes encoding heat shock proteins and a general reduction in anabolic activity [14, 15]. These biphasic response patterns reflect the need for stress protection, whilst concurrently preventing stronger negative effects on the energy balance of the organisms and cells. In this context, however, it has to be considered that mRNA expression does not necessarily imply changes in protein level [16]. In contrast to the frequently detectable correlation between increases in mRNA and protein level, decreases in the mRNA level may only serve to redirect the ribosomal machinery to other transcripts [17], with the level of corresponding proteins not much affected by these decreases.

Previous studies have focused on molecular, biochemical, physiological, and ecological aspects of the effects of temperature on *Daphnia* [10, 18, 19]. The present study aimed to analyze and compare the genome-wide responses of two different *D. pulex* genotypes (clones) to changes in temperature and/or the food supply to differentiate between the roles of different environmental impacts or clonal properties for gene regulatory adjustments in this species. The assembled and annotated draft genome sequence of *D. pulex* [20] allowed measurements of gene expression via transcriptome and proteome analyses using a microarray-based approach or 2D gel electrophoresis and mass spectrometry. These studies were conducted using an already previously investigated *D. pulex* clone (clone G) [21] and another more heat-tolerant *D. pulex* clone (clone M). First, we explored the transcriptomic response of clone G to acute heat stress by identifying differentially expressed genes (30 °C vs. 20 °C) after different periods of time (2, 4, and 8 h). Second, we investigated the chronic effects of three temperatures within the natural range of these animals (10 °C, 20 °C, and 24 °C) on differential gene expression by contrasting the corresponding transcriptomes (20 °C vs. 10 °C, 24 °C vs. 20 °C, and 24 °C vs. 10 °C acclimation). Third, we determined whether the proteomic responses to acute stress were clone-specific. Therefore, we studied protein expression in the *D. pulex* clones G and M after 24 h and 48 h under acute heat, starvation, and heat-and-starvation stress or control conditions and contrasted stress (30 °C, ad libitum feeding; starvation, 20 °C; 30 °C, starvation) and control conditions (20 °C, ad libitum feeding). The overall results of this study provide detailed information about the effects of different temporal courses and levels of environmental factors and differing clonal properties on the molecular responses of *D. pulex* to environmental changes that allow this species to cope with temperature changes and differences in the food supply.

## Results

### Transcriptomic responses of the *D. pulex* clone G to acute heat stress and temperature acclimation

#### Main features of the transcriptomic responses

Microarrays were used to study differential gene expression among four biological replicates under acute heat stress (30 °C vs. 20 °C; exposure times: 2, 4, and 8 h) or temperature acclimation (20 °C vs. 10 °C, 24 °C vs. 20 °C, 24 °C vs. 10 °C) in the *D. pulex* clone G. Genes with a false discovery rate (Q value) below 5% (0.05) were denoted as differentially expressed genes (DEGs). DEGs were identified and functionally characterized by orthology (KOG database).

**Fig. 1:**
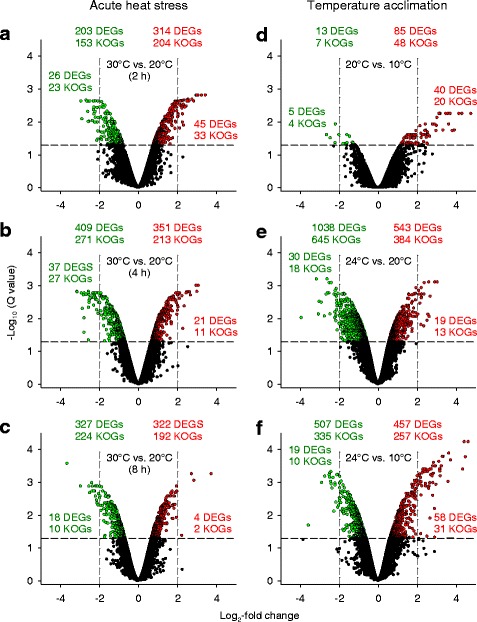
Volcano plots of the transcriptional responses of clone G to temperature changes. Contrasts of the transcriptional responses of the *D. pulex* clone G to (**a-c**) 2, 4, and 8 h under acute heat stress and control conditions (30 °C vs. 20 °C) or (**d-f**) temperature acclimation (20 °C vs. 10 °C, 24 °C vs. 20 °C, 24 °C vs. 10 °C) were depicted as statistical significance levels (−log_10_ Q values) versus expression changes (log_2_-fold changes). The green or red numbers on top are numbers of down- or upregulated differentially expressed genes (DEGs), which are defined by a Q (FDR) value ≤0.05 (the horizontal dashed lines show the position of -log_10_ (0.05) on the Y axis). The numbers directly below are numbers of KOG-identified DEGs (KOGs). The green and red numbers even lower down, which are on the left or right of the vertical dashed lines, are numbers of DEGs and KOG-identified DEGs with log_2_-fold changes < − 2 or > 2. For all experimental conditions, four biological replicates (50 animals each) were used

**Fig. 2:**
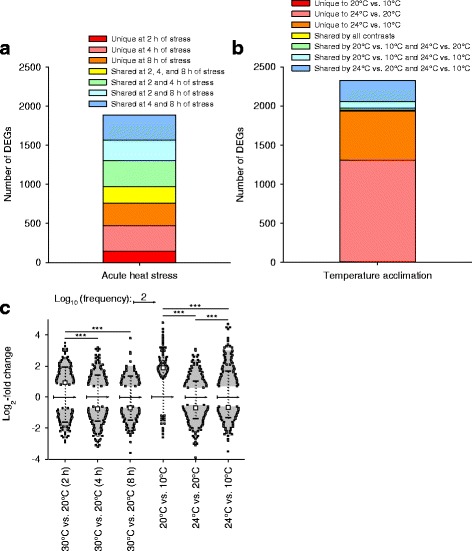
Stacked bar plots and violin plot of the transcriptional responses of clone G to temperature changes. Stacked bar plots show the number of DEGs unique to each contrast or shared by the different contrasts (**a**) from acute heat stress or (**b**) temperature acclimation. **c** The violin plot shows the frequency distribution (x-axis; log_10_ scale) of the log_2_-fold changes (y-axis) of the DEGs from the three contrasts (2, 4, and 8 h) between acute heat stress and control conditions (30 °C vs. 20 °C) and the three contrasts between different acclimation temperatures (20 °C vs. 10 °C, 24 °C vs. 20 °C, 24 °C vs. 10 °C) plus markers for the medians (squares) and the 10 and 90% quantiles (horizontal lines) of these data. Arrows indicate log_10_ scales of 2 (i.e., 100 on a linear scale) for the frequency distributions. Mann-Whitney Rank Sum tests revealed significant differences between the medians of the contrasts after 2 h and 4 or 8 h of acute heat stress as well as between the medians of all contrasts under temperature acclimation (^***^P ≤ 0.001)

**Fig. 3:**
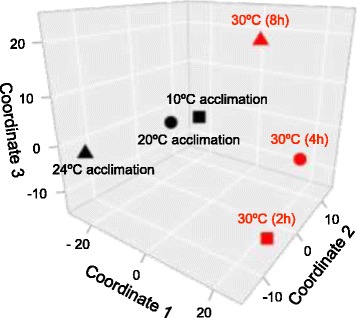
Multidimensional scaling (MDS) analysis of the transcriptional responses of clone G to temperature changes. A non-metric MDS analysis was applied to obtain a global view of similarities or dissimilarities in the transcriptional responses of the *D. pulex* clone G to temperature changes. For this purpose, the Euclidian distance between all transcriptomic data (i.e., transcriptional responses, which were normalized to either 20 °C acclimation or 20 °C controls) were scaled to three dimensions. Coordinate 1 of the MDS analysis shows a clear separation between the transcriptional responses to acute heat stress and temperature acclimation. The transcriptional responses to 2, 4, and 8 h of heat stress were different (coordinates 2 and 3) such as the responses to 10 °C, 20 °C, and 24 °C acclimation (coordinate 2)

From the 24,679 genes represented on the microarray, between 85 and 543 DEGs (48-384 KOG-identified DEGs, KOGs) were upregulated, and 13-1038 DEGs (7-645 KOGs) were downregulated upon any type of temperature change (Fig. 1). Log_2_-fold changes greater than 2 or lesser than − 2 were found for 4-58 DEGs (2-33 KOGs) or 5-37 DEGs (4-27 KOGs). Upregulated or downregulated DEGs (KOGs) dominated after 2 h or 4 h of acute heat stress (Fig. 1a, b) and in the two contrasts 20 °C vs. 10 °C or 24 °C vs. 20 °C acclimation, where the number of DEGs (KOGs) was minimum or maximum (Fig. 1d, e). Stacked bar plots show the number of DEGs unique to each contrast or shared by the different contrasts from acute heat stress (Fig. 2a) or temperature acclimation (Fig. 2b). A violin plot (Fig. 2c) shows the frequency distribution (probability density) (x-axis; log_10_ scale) of the expression changes (y-axis; log_2_-fold changes) of the DEGs from each contrast (plus markers for the medians and the 10 and 90% quantiles). The medians of the contrasts 30 °C vs. 20 °C (2 h of acute heat stress) or 20 °C vs. 10 °C acclimation were significantly higher (more positive) than the medians of the other two corresponding contrasts from acute or chronic temperature changes. A multidimensional scaling (MDS) analysis (Fig. 3) revealed no connection between the gene expression changes under acute heat stress and temperature acclimation (coordinate 1). The gene expression changes also differed among the three acclimation temperatures (coordinate 2) and the three periods of acute heat stress (coordinates 2 and 3).

#### Functional analysis of the transcriptomic responses

**Fig. 4:**
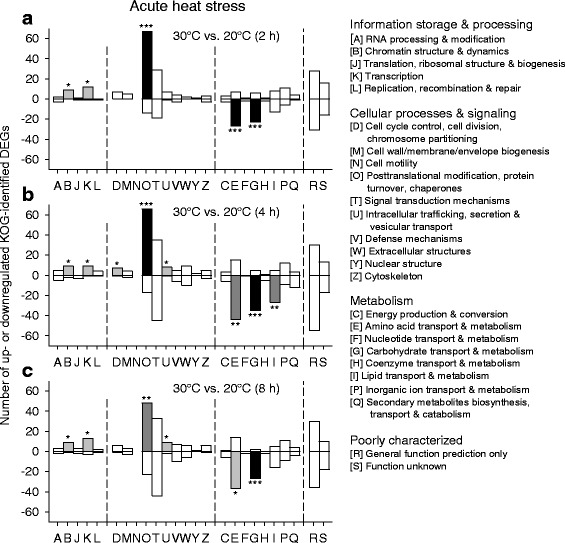
Classification of KOG-identified DEGs into KOG categories under acute heat stress. KOG-identified DEGs from the (**a**-**c**) three contrasts (2, 4, and 8 h) between acute heat stress and control conditions (30 °C vs. 20 °C) (see Fig. 1a-c) were classified into the 25 superordinated KOG categories (right). Gene enrichment analyses (chi-square tests) identified KOG categories with deviating regulation, for which the category-specific ratio between upregulated (+) and downregulated (−) DEGs was significantly different from the contrast-specific ratio (^***^P ≤ 0.001, black bars; ^**^P ≤ 0.01, dark-gray bars; ^*^P ≤ 0.05, light-gray bars). (**a**-**c**) The KOG categories O, E, and G exhibited highly significant and persistent deviations in regulation under acute heat stress

DEGs that were identified and functionally characterized by orthology (KOG-identified DEGs) accounted for about 64% of all DEGs (Fig. 1). The KOG-identified DEGs were classified for each contrast into 25 superordinated KOG categories, and gene enrichment analyses (chi-square analyses) were carried out to identify KOG categories with deviating regulation, for which the category-specific ratio between upregulated (+) and downregulated (−) DEGs was significantly different from the contrast-specific ratio (black and gray bars; Figs. 4, 6).

**Fig. 5:**
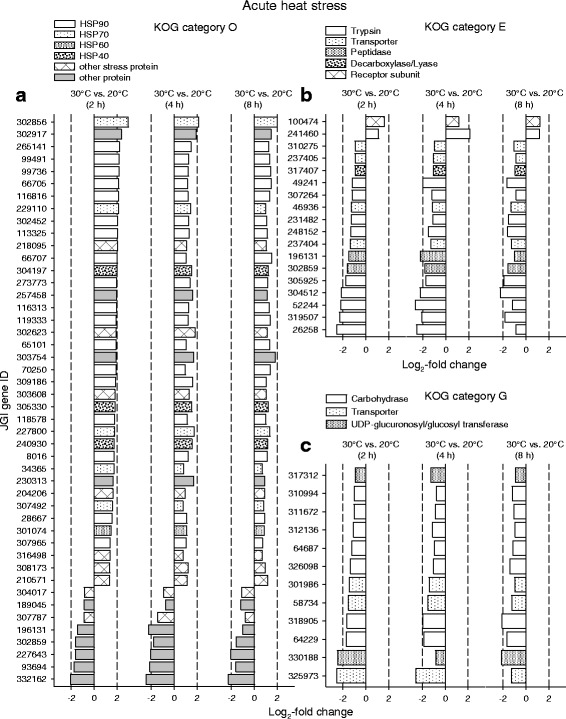
Groups of KOG-identified DEGs similarly regulated during all three periods of acute heat stress. Prominent groups of DEGs from the KOG categories O, E, and E (see Fig. 4a-c) were similarly regulated during all three periods of acute heat stress. Mostly, they encode (**a**) heat shock and stress proteins, (**b**) trypsins, transporters, and other peptidases, and (**c**) carbohydrases, transporters, and UDP-glucuronosyl/−glucosyl transferases (see also Fig. 8b and Additional file 1: Table S1). Dashed vertical lines indicate log_2_-fold changes of − 2 and 2

The KOG categories O, E, and G exhibited highly significant and persistent deviations in regulation under acute heat stress (Fig. 4a-c). Category O included numerous mostly upregulated DEGs for different types of heat shock proteins or other proteins likely involved in stress responses (e.g., FK506 binding protein/FKBP-type peptidyl-prolyl cis-trans isomerases [22], glutathione S-transferases) (Fig. 5a). Category E included mostly downregulated DEGs for trypsins, other peptidases, or transporters (Fig. 5b), and category G contained downregulated DEGs for carbohydrases, transporters, or UDP-glucuronosyl/−glucosyl transferases (Fig. 5c).

**Fig. 6:**
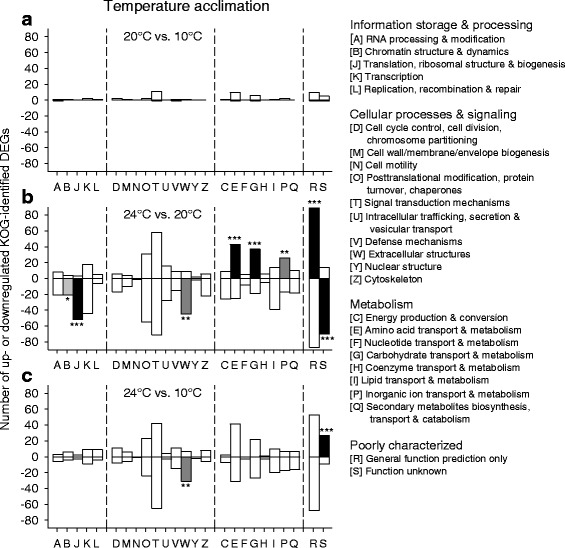
Classification of KOG-identified DEGs into KOG categories under temperature acclimation. KOG-identified DEGs from the (**a**-**c**) three contrasts between different acclimation temperatures (see Fig. 1d-f) were classified into the 25 superordinated KOG categories (right). Gene enrichment analyses (chi-square tests) identified KOG categories with deviating regulation, for which the category-specific ratio between upregulated (+) and downregulated (−) DEGs was significantly different from the contrast-specific ratio (^***^P ≤ 0.001, black bars; ^**^P ≤ 0.01, dark-gray bars; ^*^P ≤ 0.05, light-gray bars). **b** The KOG categories J, W, E, G, and P exhibited highly significant deviations in regulation in the contrast 24 °C vs. 20 °C acclimation

**Fig. 7:**
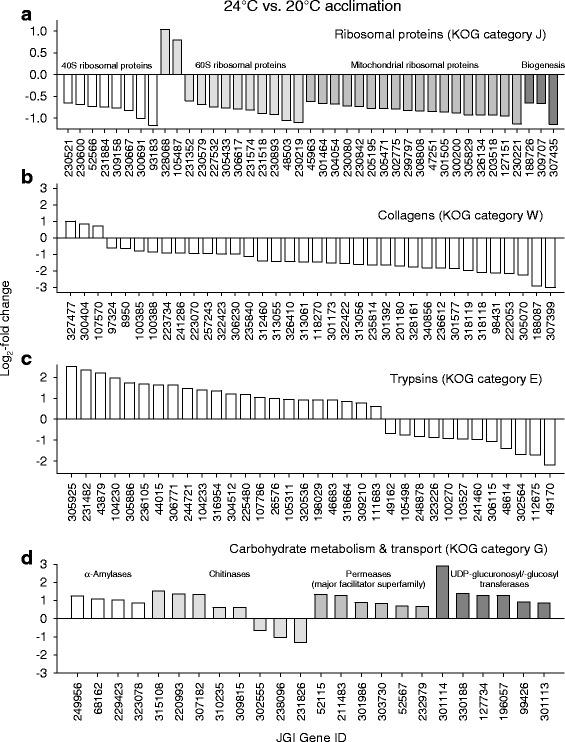
Groups of KOG-identified DEGs similarly regulated in the contrast 24 °C vs. 20 °C acclimation. Prominent groups of DEGs from the KOG categories J, W, E, and G (see Fig. 6b) were similarly regulated in the contrast 24 °C vs. 20 °C acclimation. They encode (**a**) ribosomal proteins, (**b**) collagens, (**c**) trypsins, and (**d**) carbohydrases, transporters, and UDP-glucuronosyl/−glucosyl transferases

KOG categories with deviating regulation were absent in the contrast 20 °C vs. 10 °C acclimation (Fig. 6a), where the number of DEGs was minimum (Fig. 1d). The KOG categories T, E, and G contained a few DEGs encoding, for instance, neurexin IV, trypsin, or carbohydrases. However, there were several KOG categories with highly significant deviations in regulation (categories J, W, E, G, P) in the contrast 24 °C vs. 20 °C acclimation (Fig. 6b). The categories R and S, which contained poorly characterized DEGs, were not specifically analyzed. Category J comprised mostly downregulated DEGs for ribosomal proteins (Fig. 7a), tRNA synthetases, and translation initiation factors. Category W contained many mostly downregulated DEGs for collagens type IV and XIII (Fig. 7b). Category E comprised frequently upregulated DEGs for trypsins (Fig. 7c). Prominent elements of category G were mostly upregulated DEGs for carbohydrases, permeases, and UDP-glucuronosyl/−glucosyl transferases (Fig. 7d). Many frequently upregulated DEGs of category P encode Fe^2+^-transport proteins and other primary or secondary transporters. The highly regulated DEGs of category T encode, inter alia, Ca^2+^-dependent enzymes, C-type lectins, and membrane receptors.

#### Congruent sets of DEGs

**Fig. 8:**
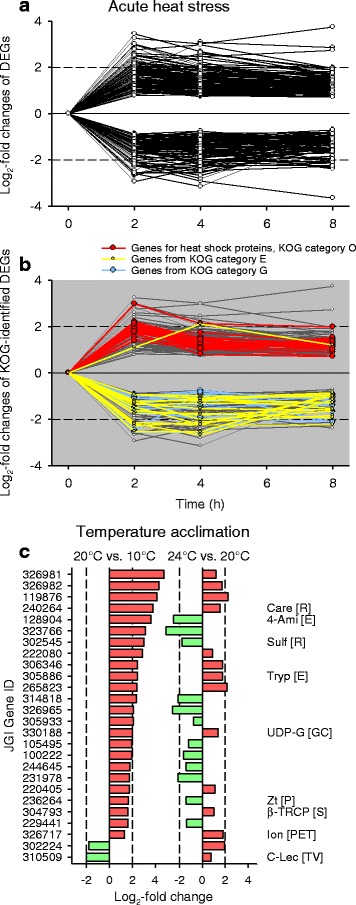
DEGs shared by all contrasts under acute heat stress or shared by 20 °C vs. 10 °C and 24 °C vs. 20 °C acclimation. There were (**a**) 217 DEGs and (**b**) 158 KOG-identified DEGs shared by all contrasts (30 °C vs. 20 °C) after 2, 4, and 8 h of acute heat stress. The KOG-identified DEGs included a high number of genes from the KOG categories O (e.g., genes for molecular chaperones, red lines), E (yellow lines), and G (blue lines). **c** There were 26 identical DEGs between 20 °C vs. 10 °C and 24 °C vs. 20 °C acclimation. They were mostly upregulated (red bars) in the contrast 20 °C vs. 10 °C acclimation. Dashed vertical lines indicate log_2_-fold changes of − 2 and 2. Abbreviations (associated KOG categories in square brackets): 4-aminobutyrate aminotransferase (4-Ami), β-transducin repeat-containing protein (β-TrCP), carboxylesterase (Care), C-type lectin (C-Lec), glutamate-gated kainate-type ion channel receptor subunit GluR5 (Ion), sulfotransferase (Sulf), trypsin (Tryp), UDP-glucuronosyl/−glucosyltransferase (UDP-G), zinc transporter (Zt)

There were 217 DEGs (Fig. 8a) and 158 KOG-identified DEGs (Fig. 8b) shared by all contrasts from acute heat stress. The functionally identified DEGs included numerous genes from the KOG categories O (e.g., genes for molecular chaperones), E (amino acid transport & metabolism), and G (carbohydate transport & metabolism). These genes are listed and disclosed in Additional file 1: Table S1.

Twenty-six DEGs were identical in the contrasts 20 °C vs. 10 °C and 24 °C vs. 20 °C acclimation (Fig. 8c). Their functions are partly unknown or quite diverse (e.g., DEGs for 1 β-transducin repeat-containing protein (β-TrCP), 1 carboxylesterase, 1 trypsin, and 1 UDP-glucuronosyl/−glucosyl transferase).

**Fig. 9:**
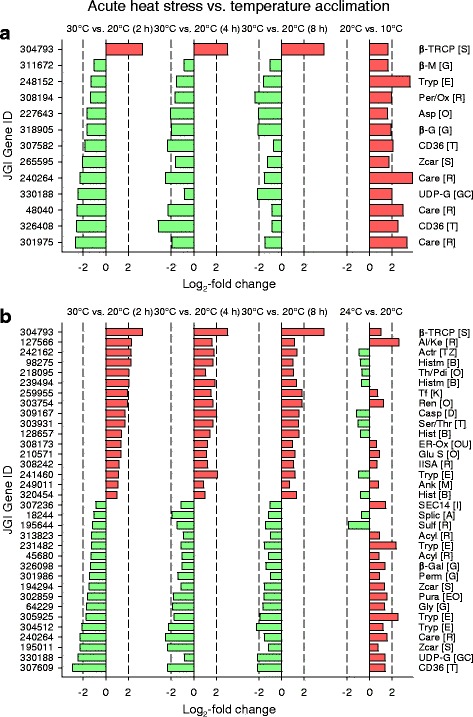
DEGs shared by all contrasts under acute heat stress and by 20 °C vs. 10 °C or 24 °C vs. 20 °C acclimation. There were 13 or 34 identical DEGs among the three periods of acute heat stress and (**a**) 20 °C vs. 10 °C acclimation or (**b**) 24 °C vs. 20 °C acclimation. The 13 identical DEGs were mostly downregulated (green bars) under acute heat stress and consistently upregulated (red bars) in the contrast 20 °C vs. 10 °C acclimation. If the 34 identical DEGs were downregulated under acute heat stress, they were frequently upregulated in the contrast 24 °C vs. 20 °C acclimation. Dashed vertical lines indicate log_2_-fold changes of − 2 and 2. See Fig. 8 for abbreviations (associated KOG categories in square brackets). Further included are actin regulatory protein (Actr), acyltransferase (Acyl), aldo/keto-reductase family (Al/Ke), alternative splicing factor SRp20/9G8 (Splic), ankyrin (Ank), aspartyl protease (Asp), β-galactosidase (β-Gal), β-glucosidase/lactase (β-G), β-mannosidase (β-M), caspase (Casp), ER-associated oxidoreductin (ER-Ox), glutathione S-transferase (Glu S), glycoprotein CD36 (CD36), glycosyl hydrolase family 31 (Gly), histone H2B (Hist), histone tail methylase (Histm), membrane protein (contains type IISA sequence) (IISA), permease (major facilitator superfamily) (Perm), peroxidase/oxygenase (Per/Ox), phosphatidylinositol transfer protein SEC14 (SEC14), puromycin-sensitive aminopeptidase (Pura), renal dipeptidase (Ren), serine/threonine protein kinase and endoribonuklease (UPR pathway) (Ser/Thr), thioredoxin/protein disulfide isomerase (Th/Pdi), transcription factor HAND2/TAL1/TAL2/LYL1 (Tf), and zinc carboxypeptidase (Zcar)

There were only 13 or 34 identical DEGs under acute heat stress (all time periods) and 20 °C vs. 10 °C acclimation (Fig. 9a) or 24 °C vs. 20 °C acclimation (Fig. 9b). The congruent 13 DEGs were mostly downregulated under acute heat stress and upregulated in case of temperature acclimation, and they encode 3 carboxylesterases, 3 proteases, 2 carbohydrases, 2 plasma membrane glycoproteins CD36, 1 β-TrCP, 1 peroxidase, and 1 UDP-glucuronosyl/−glucosyl transferase. For the congruent 34 DEGs, downregulations under acute heat stress frequently met upregulations in case of temperature acclimation. The functions of the 34 DEGs are again quite diverse (e.g., DEGs for 8 proteases, 5 stress proteins, 4 histones or histone tail methylases, 2 acyltransferases, 2 carbohydrases, 2 proteins functionally related to the cytoskeleton, 1 β-TrCP, 1 carboxylesterase, 1 permease, 1 plasma membrane glycoprotein CD36, and 1 UDP-glucuronosyl/−glucosyl transferase).

### Proteomic responses of the *D. pulex* clones G and M to acute heat, starvation, and heat-and-starvation stress

#### Preliminary investigations and principles of the proteomic studies

The stress tolerance of the *D. pulex* clones G and M was investigated by determining their mean half-maximal survival times under control conditions (20 °C, ad libitum feeding) and heat, starvation, and heat-and-starvation stress (Table 1). In comparison to control conditions, the survival times decreased severely and independently of the clone type in starving animals (starvation, 20 °C). Applying heat stress (30 °C, ad libitum feeding) or heat-and-starvation stress (30 °C, starvation) further reduced the survival times and revealed significant differences between both clones, with clone G showing significantly shorter survival times than animals of clone M.

**Table 1:** Survival times of the *D. pulex* clones G and M

	Control	30 °C, ad libitum feeding	Starvation, 20 °C	30 °C, starvation
Clone G	> 750 h	48 h	167 h	17 h
Clone M	> 750 h	98 h^***^	167 h	49 h^***^

Mean half-maximal survival times (h) of the *D. pulex* clones G and M under control conditions (20 °C, ad libitum feeding) or heat, starvation, and heat-and-starvation stress. Data are from Kaplan-Meier survival curves (*n* = 3 experiments on *N* = 10 animals each). Asterisks indicate significant differences between the clones (^***^, *P* < 0.001; Gehan-Breslow-Wilcoxon test). Clone G was more sensitive to heat or heat-and-starvation stress than clone M

Protein concentrations were measured in raw extracts of animals of the *D. pulex* clones G and M under control conditions and stress (24 and 48 h of heat, starvation, and heat-and-starvation stress) (Table 2). Protein concentrations did not differ significantly under control conditions. They did also not differ significantly between control and stress conditions in animals of clone M and between control conditions and heat or heat-and-starvation stress in animals of clone G. However, starvation stress caused decreasing protein concentrations in animals of clone G. Moreover, animals of clone G did not survive 48 h of heat-and-starvation stress. Consequently, this stress condition was not applied to animals of clone G in further experiments.

**Table 2:** Protein concentrations in the *D. pulex* clones G and M

Clone	Stress	Time (h)	Protein concentration
G	Control	0	24.3 ± 5.2
G	30 °C, ad libitum feeding	24	25.0 ± 5.3
G	Starvation, 20 °C	24	14.9 ± 2.1^*^
G	30 °C, starvation	24	16.6 ± 4.7
G	30 °C, ad libitum feeding	48	24.0 ± 4.2
G	Starvation, 20 °C	48	12.0 ± 1.8^**^
G	30 °C, starvation	48	–
M	Control	0	25.6 ± 3.8
M	30 °C, ad libitum feeding	24	25.0 ± 5.0
M	Starvation, 20 °C	24	18.0 ± 16.7
M	30 °C, starvation	24	23.0 ± 5.1
M	30 °C, ad libitum feeding	48	22.1 ± 4.7
M	Starvation, 20 °C	48	21.0 ± 3.1
M	30 °C, starvation	48	22.2 ± 6.6

Protein concentration (μg/mg fresh weight; mean ± standard deviation; per clone, stress condition, and period, *n* = 3-10 measurements on *N* = 25-30 animals each) in control animals or differently stressed animals (heat, starvation, heat-and-starvation) of the *D. pulex* clones G and M after different periods of stress exposure (24 h or 48 h). Asterisks indicate significant differences compared with control conditions (20 °C, ad libitum feeding): *, *P* < 0.05; **, *P* < 0.01 (t-tests)

Proteins in animals of the *D. pulex* clones G and M were quantified and identified by 2D gel electrophoresis and mass spectrometry of excised protein spots. Proteins were denoted as differentially expressed proteins (DEPs) if expression changed significantly (*P* < 0.05) between control conditions and 24 h or 48 h of heat, starvation, and heat-and-starvation stress. Fusion (averaged) images of 4-5 2D gels from animals of the *D. pulex* clones G and M under control conditions served as orientation for spot excision (Additional file 2: Figure S1). A total of 674 protein spots were detected, 95 of which showed a high staining intensity. These spots were excised and analyzed, which resulted in 78 identified proteins. The proteins in the other 17 spots either did not match the *D. pulex* database or could not be assigned to specific proteins.

#### Functional analysis of the proteomic responses

**Fig. 10:**
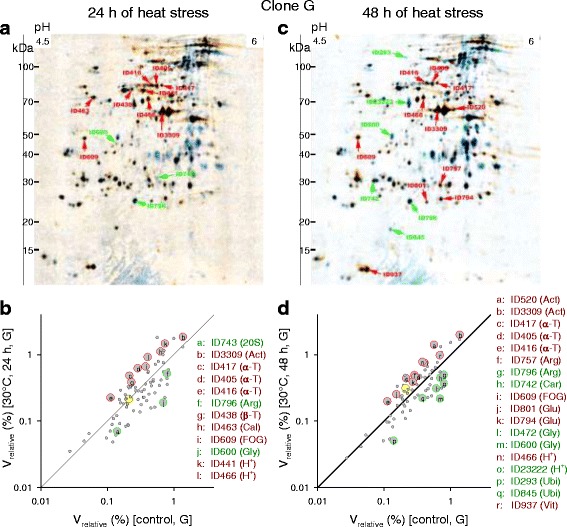
Two-dimensional protein gels from clone G under acute heat stress. The 2D gels, which are fusion (averaged) images from a varying number (*n*) of gels (biological replicates, 25-30 animals each), show changes in protein expression in the *D. pulex* clone G after the acute exposure of control animals (20 °C, ad libitum feeding) (blue spots; *n* = 5) to (**a**) 24 h (orange spots; *n* = 4) or (**c**) 48 h (orange spots; *n* = 6) of heat stress (30 °C, ad libitum feeding). Red or green spot IDs mark significantly up- or downregulated proteins (t-tests, *P* < 0.05; see Table 3). The scatter plots show changes in expression levels (V_relative_, relative spot volume) between control and heat-stressed animals (**b**, 24 h; **d**, 48 h) of significantly (large circles and letters) or non-significantly (small circles) up- or downregulated proteins (data from **a** or **c**). Proteins, which were upregulated under heat stress, are shown above the diagonal line. Spot IDs and functions are from Table 3. For comparison reasons (see Fig. 14), the expression levels of the non-significantly regulated protein ID383 (JGI gene ID: 301074, Chaperonin ATPase, Cpn60/Hsp60p; e.g., Table 3) are also shown (yellow circles). Abbreviations for Figs. 10, 11, 12, 15, and Additional files 3, 4, 5, 6: Figures S2-S5: 3-phosphoglycerate kinase (3-P), 20S proteasome (20S), actin (Act), α-tubulin (α-T), arginine kinase (Arg), β-glucosidase (β-G), β-tubulin (β-T), calreticulin (Cal), carboxypeptidase (Car), chaperonin ATPase (HSP60), cytosolic fatty-acid binding protein (Cyt), enolase (Eno), FKBP-type peptidyl-prolyl cis-trans isomerase (FKBP), FOG, leucine-rich repeat (FOG), glutathione transferase (Glu), glyceraldehyde 3-phosphate dehydrogenase (G3pd), glycoside hydrolase (Gly), H^+^-transporting two-sector ATPase (H^+^), M13 family peptidase (M13), peptidase S1 (Pep), protein disulfide isomerase (Pdi), serine endopeptidase (Ser), ubiquitin (Ubi), vitellogenin (Vit)

**Fig. 11:**
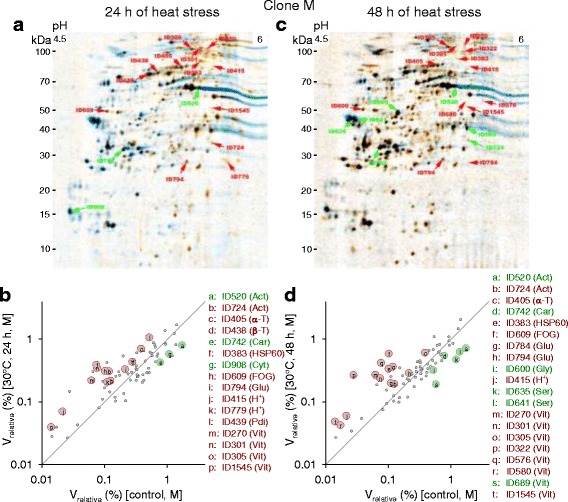
Two-dimensional protein gels from clone M under acute heat stress. The 2D gels, which are fusion (averaged) images from a varying number (*n*) of gels (biological replicates, 25-30 animals each), show changes in protein expression in the *D. pulex* clone M after the acute exposure of control animals (20 °C, ad libitum feeding) (blue spots; *n* = 4) to (**a**) 24 h (orange spots; *n* = 3) or (**c**) 48 h (orange spots; *n* = 10) of heat stress (30 °C, ad libitum feeding). **b**, **d** Spot IDs and functions are from Table 3. See Fig. 10 for further explanations

Evaluating the fusion images of 2D gels from animals of the *D. pulex* clones G and M after 24 h or 48 h of heat stress (Fig. 10a, c; Fig. 11a, c) revealed 12 or 18 DEPs in clone G (Fig. 10b, d) and 16 or 20 DEPs in clone M (Fig. 11b, d), with 22 (clone G) or 24 (clone M) proteins showing significant expression changes after some period of heat stress (24 and/or 48 h) (Table 3). The DEPs included actins, α-tubulins, glutathione transferases, glycoside hydrolases, H^+^-transporting two-sector ATPases, and vitellogenins. An arginine kinase and an ubiquitin/ubiquitin-like protein were, for instance, specific for clone G. The measured molecular masses of the ubiquitin proteins (18.3-107.3 kDa) were clearly higher than the predicted molecular mass (5.7 kDa), which indicates an attachment to unknown target proteins for the purpose of protein degradation. This assumption was supported by the presence of ubiquitin as a companion protein in other spots. A chaperonin ATPase (HSP60), a serine endopeptidase, and a vitellogenin fused to superoxide dismutase (SOD) were, inter alia, specific for clone M. The measured molecular masses (12.1-110.9 kDa) of both vitellogenin types (*D. pulex* clones G and M) were markedly lower than the predicted molecular masses (191.9-223 kDa), suggesting that protein cleavage resulted in their fragmentation.

**Table 3:** Differentially expressed proteins (DEPs) in the *D. pulex* clones G and M under heat stress

Spot ID	Gene ID	Function	R	*P*	R	*P*	SP	M_r_*e*	M_r_*p*	pI*e*	pI*p*	SC (%)	NP
			24 h		48 h								
743	306433	20S Proteasome, A and B subunits	**0.5**	**0.00**	0.7	0.14	–	34.3	28	5.3	5.3	35.9	8
520	300012	Actin and related proteins	3.1	0.06	**2.4**	**0.04**	–	62.6	41.8	5.5	5.3	30.0	8
3309	306442	Actin and related proteins	**1.3**	**0.03**	**1.4**	**0.00**	–	62.6	41.9	5.4	5.2	30.8	8
417	100611	α-Tubulin	**2.2**	**0.02**	**2.0**	**0.00**	–	82.3	46.1	5.4	5.0	23.8	8
405	301837	α-Tubulin	**2.1**	**0.00**	**1.7**	**0.01**	–	81.9	49.9	5.3	5.0	33.0	16
416	301837	α-Tubulin	**1.9**	**0.00**	**1.7**	**0.02**	–	81.9	49.9	5.2	5.0	11.8	6
757	220693	Arginine kinase	0.9	0.35	**1.5**	**0.01**	–	28.8	39.9	5.4	5.5	36.3	9
796	220693	Arginine kinase	**0.6**	**0.03**	**0.6**	**0.01**	–	24.4	39.9	5.1	5.5	34.9	10
438	300845	β-Tubulin	**1.6**	**0.01**	1.3	0.09	–	78.8	50.1	5.1	4.7	19.9	10
463	210624	Calreticulin	**1.7**	**0.03**	1.2	0.44	16	72.4	46.6	4.7	4.5	48.3	18
742	303899	Carboxypeptidase A2	0.8	0.06	**0.6**	**0.01**	17	30.0	46.1	4.8	5.2	29.6	14
609	304126	FOG, leucine-rich repeat	**1.7**	**0.00**	**2.0**	**0.00**	22	47.2	42.1	4.6	4.7	18.6	7
801	303282	Glutathione transferase	1.3	0.07	**1.6**	**0.00**	–	24.2	24.8	5.3	5.4	36.8	12
794	317266	Glutathione transferase	1.0	0.84	**1.4**	**0.05**	–	25.0	25	5.4	5.2	67.3	18
472	300366	Glycoside hydrolase, family 7	0.5	0.07	**0.5**	**0.02**	19	70.2	50.3	4.8	4.8	21.5	8
600	303,036	Glycoside hydrolase, family 16	**0.3**	**0.01**	**0.3**	**0.00**	19	47.5	40.4	4.9	4.8	30.1	12
441	309746	H^+^-transporting two-sector ATPase	**1.9**	**0.00**	1.4	0.07	–	78.1	56.8	5.2	5.5	43.8	16
466	309746	H^+^-transporting two-sector ATPase	**2.2**	**0.00**	**1.8**	**0.00**	–	71.4	56.8	5.2	5.5	79.4	56
23,222	309746	H^+^-transporting two-sector ATPase	0.5	0.09	**0.5**	**0.02**	–	66.8	56.8	5.1	5.5	65.5	23
293	9558	Ubiquitin and ubiquitin-like proteins	0.8	0.63	**0.4**	**0.04**	–	107.3	5.7	5.1	5.2	35.3	2
845	9558	Ubiquitin and ubiquitin-like proteins	0.9	0.59	**0.6**	**0.01**	–	18.3	5.7	4.9	5.2	35.3	2
937	308693	Vitellogenin	1.4	0.08	**1.4**	**0.05**	20	12.1	191.9	4.7	6.5	11.3	16
Spot ID	Gene ID	Function	R	*P*	R	*P*	SP	M_r_*e*	M_r_*p*	pI*e*	pI*p*	SC (%)	NP
			24 h		48 h								
520	300012	Actin and related proteins	**0.5**	**0.04**	**0.4**	**0.00**	–	62.6	41.8	5.5	5.3	30.0	8
724	305550	Actin and related proteins	**1.7**	**0.02**	**1.5**	**0.04**	–	32.0	41.8	5.7	5.4	37.9	12
405	301837	α-Tubulin	**1.8**	**0.01**	**1.5**	**0.01**	–	81.9	49.9	5.3	5.0	33.0	16
438	300,845	β-Tubulin	**1.5**	**0.01**	1.0	0.80	–	78.8	50.1	5.1	4.7	19.9	10
742	303899	Carboxypeptidase A2	**0.5**	**0.03**	**0.7**	**0.05**	17	30.0	46.1	4.8	5.2	29.6	14
383	301074	Chaperonin ATPase, Cpn60/Hsp60p	**5.1**	**0.01**	**3.1**	**0.04**	–	89.1	61.4	5.6	5.8	31.2	22
908	300446	Cytosolic fatty-acid binding protein	**0.5**	**0.04**	0.8	0.25	–	26.1	14.8	6.0	5.3	31.1	4
609	304126	FOG, leucine-rich repeat	**3.0**	**0.00**	**5.6**	**0.00**	22	47.2	42.1	4.6	4.7	18.6	7
784	305501	Glutathione transferase	1.6	0.06	**1.8**	**0.03**	–	25.3	25.2	5.8	5.6	36.2	10
794	317266	Glutathione transferase	**1.5**	**0.03**	**1.3**	**0.02**	–	25.0	25	5.4	5.2	67.3	18
600	303036	Glycoside hydrolase, family 16	0.5	0.14	**0.6**	**0.03**	19	47.5	40.4	4.9	4.8	30.1	12
415	306451	H^+^-transporting two-sector ATPase	**3.2**	**0.03**	**2.7**	**0.02**	–	66.9	55.5	5.7	5.5	57.6	56
779	309746	H^+^-transporting two-sector ATPase	**1.8**	**0.01**	1.6	0.10	–	26.5	56.8	5.9	5.5	64.2	20
439	234212	Protein disulfide isomerase	**2.0**	**0.00**	1.3	0.23	15	78.1	58.3	5.0	4.8	44.4	22
635	251885	Serine endopeptidase	0.4	0.16	**0.4**	**0.01**	19	41.5	39.6	4.4	5.0	12.7	6
641	251885	Serine endopeptidase	0.6	0.21	**0.4**	**0.01**	19	41.2	39.6	4.5	5.0	12.7	6
270	219769	Vitellogenin fused with SOD	**3.4**	**0.05**	**4.2**	**0.02**	17	110.9	223	5.6	6.5	12.3	22
301	219769	Vitellogenin fused with SOD	**4.4**	**0.02**	**5.3**	**0.00**	17	107.0	223	5.6	6.5	13.3	34
305	219769	Vitellogenin fused with SOD	**2.5**	**0.04**	**3.0**	**0.00**	17	104.8	223	5.5	6.5	12.5	23
322	219769	Vitellogenin fused with SOD	5.1	0.06	**9.3**	**0.01**	17	102.6	223	5.6	6.5	12.3	21
576	219769	Vitellogenin fused with SOD	1.6	0.31	**3.2**	**0.05**	17	51.9	223	5.7	6.5	7.5	18
580	219769	Vitellogenin fused with SOD	2.2	0.13	**2.4**	**0.04**	17	51.8	223	5.6	6.5	9.9	22
689	219769	Vitellogenin fused with SOD	0.9	0.87	**0.3**	**0.01**	17	38.1	223	5.7	6.5	12.9	25
1545	219769	Vitellogenin fused with SOD	**2.7**	**0.05**	**3.3**	**0.01**	17	51.8	223	5.6	6.5	9.9	22

*D. pulex* clone G on top, *D. pulex* clone M at the bottom. From left to right: Spot identity (ID), gene ID (JGI_V11_gene ID), protein function (described in the protein sequence database of the *Daphnia pulex* genome assembly v1.0), expression ratios (R) contrasting stress (24 h or 48 h at 30 °C, ad libitum feeding) and control (20 °C, ad libitum feeding) conditions and associated *P* values (t-tests), predicted length of N-terminal signal peptides (SP), experimental and predicted molecular masses (M_r_*e*, M_r_*p*) and isoelectric points (pI*e*, pI*p*) of the mature protein (without signal peptide), sequence coverage (SC; percentage of the predicted protein sequence covered by matching peptide sequences), and number of matching peptides (NP). Bold R and *P* values indicate statistical significance. Number of gels (biological replicates, 25-30 animals each) for clone G or M: control (5 or 4) and 24 h (4 or 3) or 48 h (6 or 10) of stress

Analyzing the fusion images of 2D gels from animals of the *D. pulex* clones G and M after 24 h or 48 h of starvation stress (Additional file 3: Figure S2a, c; Additional file 4: Figure S3a, c) revealed 9 or 12 DEPs in clone G (Additional file 3: Figure S2b, d) and 22 or 21 DEPs in clone M (Additional file 4: Figure S3b, d), with 19 (clone G) or 31 (clone M) proteins showing significant expression changes after some period of starvation stress (24 and/or 48 h) (Table 4). The DEPs included actins, α-tubulins, an arginine kinase, glutathione transferases, an H^+^-transporting two-sector ATPase, and a vitellogenin fused to SOD. An HSP60 and an enolase were, inter alia, specific for clone M.

**Table 4:** Differentially expressed proteins (DEPs) in the *D. pulex* clones G and M under starvation stress

Spot ID	Gene ID	Function	R	*P*	R	*P*	SP	M_r_*e*	M_r_*p*	pI*e*	pI*p*	SC (%)	NP
			24 h		48 h								
501	299795	3-Phosphoglycerate kinase	1.6	0.12	**2.2**	**0.00**	–	83.4	44.2	5.6	5.5	39.4	28
550	300012	Actin and related proteins	1.4	0.08	**0.6**	**0.02**	–	60.5	41.8	5.3	5.3	14.3	4
752	300012	Actin and related proteins	**2.7**	**0.04**	1.0	0.96	–	28.2	41.8	5.6	5.3	36.3	17
749	305550	Actin and related proteins	1.8	0.18	**0.5**	**0.02**	–	29.1	41.8	5.5	5.3	28.9	10
3309	306442	Actin and related proteins	1.1	0.30	**1.8**	**0.01**	–	62.6	41.9	5.4	5.2	30.8	8
417	100611	α-Tubulin	1.4	0.16	**1.5**	**0.01**	–	82.3	46.1	5.4	5.0	23.8	8
405	301837	α-Tubulin	1.0	0.95	**1.5**	**0.03**	–	81.9	49.9	5.3	5.0	33.0	16
416	301837	α-Tubulin	0.8	0.17	**1.6**	**0.04**	–	81.9	49.9	5.2	5.0	11.8	6
543	220693	Arginine kinase	1.7	0.09	**2.6**	**0.00**	–	57.6	39.9	5.7	5.5	58.4	32
757	220693	Arginine kinase	**1.8**	**0.04**	0.8	0.06	–	28.8	39.9	5.4	5.5	36.3	9
463	210624	Calreticulin	**0.6**	**0.05**	0.7	0.06	16	72.4	46.6	4.7	4.5	48.3	18
742	303899	Carboxypeptidase A2	**0.6**	**0.03**	0.9	0.31	17	30.0	46.1	4.8	5.2	29.6	14
908	300446	Cytosolic fatty-acid binding protein	1.0	0.93	**0.3**	**0.03**	–	26.1	14.8	6.0	5.3	31.1	4
805	231271	FKBP-type peptidyl-prolyl cis-trans isomerase	0.9	0.81	**0.6**	**0.03**	17	23.3	23.4	4.5	4.5	66.7	14
794	317266	Glutathione transferase	**1.7**	**0.02**	**1.2**	**0.03**	–	25.0	25	5.4	5.2	67.3	18
779	309746	H^+^-transporting two-sector ATPase	**1.8**	**0.04**	1.8	0.07	–	26.5	56.8	5.9	5.5	64.2	20
722	248155	Peptidase S1, chymotrypsin	**2.5**	**0.04**	**1.7**	**0.02**	15	39.9	30.2	5.5	4.7	22.3	3
845	9558	Ubiquitin and ubiquitin-like proteins	**0.7**	**0.04**	0.7	0.08	–	18.3	5.7	4.9	5.2	35.3	2
689	219769	Vitellogenin fused with SOD	**2.2**	**0.04**	1.9	0.06	17	38.1	223	5.7	6.5	12.9	25
Spot ID	Gene ID	Function	R	*P*	R	*P*	SP	M_r_*e*	M_r_*p*	pI*e*	pI*p*	SC (%)	NP
			24 h		48 h								
520	300012	Actin and related proteins	**0.2**	**0.00**	**0.3**	**0.00**	–	62.6	41.8	5.5	5.3	30.0	8
550	300012	Actin and related proteins	**1.8**	**0.03**	**1.8**	**0.01**	–	60.5	41.8	5.3	5.3	14.3	4
698	300012	Actin and related proteins	**1.8**	**0.01**	**2.0**	**0.00**	–	36.2	41.8	5.1	5.3	22.8	8
752	300012	Actin and related proteins	**4.4**	**0.00**	**3.5**	**0.03**	–	28.2	41.8	5.6	5.3	36.3	17
724	305550	Actin and related proteins	1.7	0.06	**2.2**	**0.02**	–	32.0	41.8	5.7	5.3	37.9	12
749	305550	Actin and related proteins	**3.7**	**0.00**	1.9	0.10	–	29.1	41.8	5.5	5.3	28.9	10
417	100611	α-Tubulin	**0.6**	**0.02**	**0.7**	**0.04**	–	82.3	46.1	5.4	5.0	23.8	8
405	301837	α-Tubulin	**0.6**	**0.03**	0.9	0.45	–	81.9	49.9	5.3	5.0	33.0	16
757	220693	Arginine kinase	**2.5**	**0.01**	1.7	0.06	–	28.8	39.9	5.4	5.5	36.3	9
796	220693	Arginine kinase	**2.1**	**0.03**	1.6	0.08	–	24.4	39.9	5.1	5.5	34.9	10
438	300845	β-Tubulin	**0.6**	**0.01**	0.9	0.72	–	78.8	50.1	5.1	4.7	19.9	10
463	210624	Calreticulin	**0.7**	**0.03**	**0.6**	**0.02**	16	72.4	46.6	4.7	4.5	48.3	18
383	301074	Chaperonin ATPase, Cpn60/Hsp60p	**3.8**	**0.01**	**2.9**	**0.00**	–	89.1	61.4	5.6	5.8	31.2	22
809	301844	Enolase	**1.9**	**0.00**	1.4	0.42	–	26.1	46.8	6.0	5.7	28.1	8
1027	301844	Enolase	**1.9**	**0.04**	1.9	0.26	–	5.0	46.8	6.6	5.7	19.8	6
609	304126	FOG, leucine-rich repeat	1.5	0.07	**2.9**	**0.01**	22	47.2	42.1	4.6	4.7	18.6	7
801	303282	Glutathione transferase	**1.8**	**0.01**	1.4	0.12	–	24.2	24.8	5.3	5.4	36.8	12
784	305501	Glutathione transferase	1.3	0.16	**2.5**	**0.04**	–	25.3	25.2	5.8	5.6	36.2	10
794	317266	Glutathione transferase	**1.7**	**0.01**	1.3	0.27	–	25.0	25	5.4	5.2	67.3	18
474	230437	Glycoside hydrolase, family 9	**0.2**	**0.02**	**0.4**	**0.02**	18	71.5	49.1	5.3	5.2	18.2	6
441	309746	H^+^-transporting two-sector ATPase	**0.5**	**0.03**	0.8	0.34	–	78.1	56.8	5.2	5.5	43.8	16
779	309746	H^+^-transporting two-sector ATPase	1.1	0.73	**3.0**	**0.03**	–	26.5	56.8	5.9	5.5	64.2	20
641	251885	Serine endopeptidase	0.9	0.65	**0.4**	**0.03**	19	41.2	39.6	4.5	5.0	12.7	6
845	9558	Ubiquitin and ubiquitin-like proteins	0.7	0.32	**0.6**	**0.04**	–	18.3	5.7	4.9	5.2	35.3	2
301	219769	Vitellogenin fused with SOD	**1.6**	**0.04**	**2.2**	**0.03**	17	107.0	223	5.6	6.5	13.3	34
322	219769	Vitellogenin fused with SOD	**2.3**	**0.03**	**2.8**	**0.03**	17	102.6	223	5.6	6.5	12.3	21
325	219769	Vitellogenin fused with SOD	**2.5**	**0.01**	**2.6**	**0.04**	17	101.9	223	5.6	6.5	12.6	29
576	219769	Vitellogenin fused with SOD	1.7	0.15	**2.8**	**0.01**	17	51.9	223	5.7	6.5	7.5	18
580	219769	Vitellogenin fused with SOD	5.1	0.05	**2.9**	**0.01**	17	51.8	223	5.6	6.5	9.9	22
660	219769	Vitellogenin fused with SOD	1.1	0.70	**2.4**	**0.00**	17	40.0	223	5.3	6.5	12.8	26
1545	219769	Vitellogenin fused with SOD	**4.7**	**0.00**	**5.0**	**0.00**	17	51.8	223	5.6	6.5	9.9	22

*D. pulex* clone G on top, *D. pulex* clone M at the bottom. From left to right: Spot ID, gene ID, protein function, expression ratios (R) contrasting stress (24 h or 48 h under starvation, 20 °C) and control (20 °C, ad libitum feeding) conditions and associated *P* values (t-tests), predicted length of N-terminal signal peptides (SP), experimental and predicted molecular masses (M_r_*e*, M_r_*p*) and isoelectric points (pI*e*, pI*p*) of the mature protein, sequence coverage (SC), and number of matching peptides (NP). Bold R and *P* values indicate statistical significance. See Table 3 for details. Number of gels (biological replicates, 25-30 animals each) for clone G or M: control (5 or 4) and 24 h (4 or 4) or 48 h (4 or 5) of stress

Evaluating the fusion images of 2D gels from animals of the *D. pulex* clones G and M after 24 h or 48 h of heat-and-starvation stress (Additional file 5: Figure S4a; Additional file 6: Figure S5a, c) revealed 16 DEPs in clone G (Additional file 5: Figure S4b) and 23 or 38 DEPs in clone M (Additional file 6: Figure S5b, d), with 16 (clone G) or 41 (clone M) proteins showing significant expression changes after some period of heat-and-starvation stress (24 and/or 48 h) (Table 5). The DEPs included actins, an arginine kinase and an HSP60, glutathione transferases, H^+^-transporting two-sector ATPases, an ubiquitin/ubiquitin-like protein, and vitellogenins. An enolase and a serine endopeptidase were, inter alia, specific for clone M.

**Table 5:** Differentially expressed proteins (DEPs) in the *D. pulex* clones G and M under heat-and-starvation stress

Spot ID	Gene ID	Function			R	*P*	SP	M_r_*e*	M_r_*p*	pI*e*	pI*p*	SC (%)	NP
					24 h								
501	299795	3-Phosphoglycerate kinase			**1.9**	**0.00**	–	83.4	44.2	5.6	5.5	39.4	28
520	300012	Actin and related proteins			**1.8**	**0.05**	–	62.6	41.8	5.5	5.3	30.0	8
3309	306442	Actin and related proteins			**1.4**	**0.02**	–	62.6	41.9	5.4	5.2	30.8	8
543	220693	Arginine kinase			**2.0**	**0.03**	–	58.0	39.9	5.6	5.5	58.4	32
563	220693	Arginine kinase			**3.1**	**0.03**	–	57.6	39.9	5.6	5.5	58.4	32
796	220693	Arginine kinase			**0.5**	**0.03**	–	24.4	39.9	5.1	5.5	34.9	10
742	303899	Carboxypeptidase A2			**0.6**	**0.02**	17	30.0	46.1	4.8	5.2	29.6	14
383	301074	Chaperonin ATPase, Cpn60/Hsp60p			**1.8**	**0.03**	–	89.1	61.4	5.6	5.8	31.2	22
801	303282	Glutathione transferase			**1.6**	**0.00**	–	24.2	24.8	5.3	5.4	36.8	12
784	305501	Glutathione transferase			**1.6**	**0.03**	–	25.3	25.2	5.8	5.6	36.2	10
600	303036	Glycoside hydrolase, family 16			**0.3**	**0.01**	19	47.5	40.4	4.9	4.8	30.1	12
779	309746	H^+^-transporting two-sector ATPase			**2.0**	**0.00**	–	26.5	56.8	5.9	5.5	64.2	20
23,222	309746	H^+^-transporting two-sector ATPase			**0.4**	**0.03**	–	66.8	56.8	5.1	5.5	65.5	23
722	248155	Peptidase S1, chymotrypsin			**2.0**	**0.02**	15	39.9	30.2	5.5	4.7	22.3	3
845	9558	Ubiquitin and ubiquitin-like proteins			**0.6**	**0.01**	–	18.3	5.7	4.9	5.2	35.3	2
448	308693	Vitellogenin			**0.5**	**0.01**	20	74.3	191.9	5.5	6.5	9.8	31
Spot ID	Gene ID	Function	R	*P*	R	*P*	SP	M_r_*e*	M_r_*p*	pI*e*	pI*p*	SC (%)	NP
			24 h		48 h								
520	300012	Actin and related proteins	**0.4**	**0.00**	**0.4**	**0.00**	–	62.6	41.8	5.5	5.3	29.97	8
752	300012	Actin and related proteins	1.0	0.73	**1.7**	**0.01**	–	28.2	41.8	5.6	5.3	36.34	17
724	305550	Actin and related proteins	**2.3**	**0.01**	**2.5**	**0.00**	–	32.0	41.8	5.7	5.3	37.93	12
3309	306442	Actin and related proteins	1.4	0.19	**1.5**	**0.03**	–	62.6	41.9	5.4	5.2	30.77	8
405	301837	α-Tubulin	**1.6**	**0.02**	**1.6**	**0.04**	–	81.9	49.9	5.3	5.0	33.04	16
543	220693	Arginine kinase	1.7	0.06	**1.9**	**0.03**	–	58.0	39.9	5.6	5.5	58.38	32
563	220693	Arginine kinase	1.4	0.11	**2.0**	**0.00**	–	57.6	39.9	5.6	5.5	58.38	32
411	314456	β-Glucosidase	0.9	0.69	**0.6**	**0.03**	19	81.9	57.1	5.0	4.9	15.54	9
463	210624	Calreticulin	0.9	0.49	**0.5**	**0.00**	16	72.4	46.6	4.7	4.5	48.3	18
742	303899	Carboxypeptidase A2	0.9	0.51	**0.5**	**0.00**	17	30.0	46.1	4.8	5.2	29.59	14
383	301074	Chaperonin ATPase, Cpn60/Hsp60p	**5.5**	**0.00**	**7.1**	**0.00**	–	89.1	61.4	5.6	5.8	31.2	22
908	300446	Cytosolic fatty-acid binding protein	0.5	0.05	**0.2**	**0.00**	–	26.1	14.8	6.0	5.3	31.06	4
809	301844	Enolase	**1.6**	**0.02**	1.5	0.26	–	26.1	46.8	6.0	5.7	28.11	8
1027	301844	Enolase	1.1	0.53	**1.4**	**0.05**	–	5.0	46.8	6.6	5.7	19.82	6
805	231271	FKBP-type peptidyl-prolyl cis-trans isomerase	**0.6**	**0.03**	0.8	0.35	17	23.3	23.4	4.5	4.5	66.67	14
609	304126	FOG, leucine-rich repeat	**3.2**	**0.02**	**3.5**	**0.00**	22	47.2	42.1	4.6	4.7	18.64	7
801	303282	Glutathione transferase	**1.7**	**0.04**	1.3	0.13	–	24.2	24.8	5.3	5.4	36.77	12
784	305501	Glutathione transferase	**3.0**	**0.00**	**3.4**	**0.00**	–	25.3	25.2	5.8	5.6	36.24	10
794	317266	Glutathione transferase	**1.6**	**0.02**	**1.6**	**0.04**	–	25.0	25	5.4	5.2	67.26	18
600	303036	Glycoside hydrolase, family 16	0.8	0.35	**0.5**	**0.01**	19	47.5	40.4	4.9	4.8	30.08	12
415	306451	H^+^-transporting two-sector ATPase	**3.2**	**0.01**	**9.1**	**0.00**	–	66.9	55.5	5.7	5.5	57.6	56
466	309746	H^+^-transporting two-sector ATPase	1.2	0.39	**1.7**	**0.02**	–	71.4	56.8	5.2	5.5	79.4	56
779	309746	H^+^-transporting two-sector ATPase	**2.9**	**0.02**	**3.1**	**0.01**	–	26.5	56.8	5.9	5.5	64.23	20
23,222	309746	H^+^-transporting two-sector ATPase	1.0	0.80	**0.4**	**0.00**	–	66.8	56.8	5.1	5.5	65.54	23
332	200882	M13 family peptidase	0.6	0.07	**0.3**	**0.00**	–	99.0	75.4	4.7	4.6	47.86	34
635	251885	Serine endopeptidase	**0.3**	**0.02**	**0.3**	**0.01**	19	41.5	39.6	4.4	5.0	12.71	6
641	251885	Serine endopeptidase	**0.2**	**0.01**	**0.3**	**0.00**	19	41.2	39.6	4.5	5.0	12.71	6
444	9558	Ubiquitin and ubiquitin-like proteins	**2.4**	**0.00**	**4.8**	**0.00**	–	76.2	5.7	4.6	5.2	35.29	2
845	9558	Ubiquitin and ubiquitin-like proteins	0.7	0.09	**0.5**	**0.00**	–	18.3	5.7	4.9	5.2	35.29	2
231	308693	Vitellogenin	3.5	0.08	**5.2**	**0.01**	20	117.0	191.9	5.7	6.5	16.34	47
238	308693	Vitellogenin	3.7	0.13	**2.9**	**0.04**	20	117.0	191.9	5.8	6.5	18.39	46
270	219769	Vitellogenin fused with SOD	**2.5**	**0.02**	**4.6**	**0.02**	17	110.9	223	5.6	6.5	12.3	22
301	219769	Vitellogenin fused with SOD	**3.9**	**0.01**	**3.9**	**0.00**	17	107.0	223	5.6	6.5	13.25	34
305	219769	Vitellogenin fused with SOD	**3.8**	**0.00**	**2.5**	**0.01**	17	104.8	223	5.5	6.5	12.45	23
322	219769	Vitellogenin fused with SOD	**3.0**	**0.01**	**3.5**	**0.00**	17	102.6	223	5.6	6.5	12.25	21
325	219769	Vitellogenin fused with SOD	**3.8**	**0.00**	**4.0**	**0.00**	17	101.9	223	5.6	6.5	12.55	29
576	219769	Vitellogenin fused with SOD	**2.4**	**0.01**	**3.6**	**0.00**	17	51.9	223	5.7	6.5	7.5	18
580	219769	Vitellogenin fused with SOD	**3.3**	**0.00**	**7.0**	**0.00**	17	51.8	223	5.6	6.5	9.85	22
616	219769	Vitellogenin fused with SOD	1.3	0.32	**2.1**	**0.01**	17	45.1	223	5.6	6.5	8.9	24
689	219769	Vitellogenin fused with SOD	0.7	0.55	**0.2**	**0.02**	17	38.1	223	5.7	6.5	12.85	25
1545	219769	Vitellogenin fused with SOD	**3.1**	**0.02**	**6.7**	**0.02**	17	51.8	223	5.6	6.5	9.85	22

*D. pulex* clone G on top, *D. pulex* clone M at the bottom. From left to right: Spot ID, gene ID, protein function, expression ratios (R) contrasting stress (24 h or 48 h at 30 °C, starvation) and control (20 °C, ad libitum feeding) conditions and associated *P* values (t-tests), predicted length of N-terminal signal peptides (SP), experimental and predicted molecular masses (M_r_*e*, M_r_*p*) and isoelectric points (pI*e*, pI*p*) of the mature protein, sequence coverage (SC), and number of matching peptides (NP). Bold R and *P* values indicate statistical significance. (Clone G did not survive 48 h of heat-and-starvation stress.) See Table 3 for details. Number of gels (biological replicates, 25-30 animals each) for clone G or M: control (5 or 4) and 24 h (5 or 5) or 48 h (0 or 7) of stress

Some common aspects of these data should be addressed here. Attachments to other proteins, protein cleavage, or post-translational modifications could explain why identical proteins were repeatedly present in different spots, and why measured (M_r_*e*) and predicted (M_r_*p*) molecular masses repeatedly deviated more severely (see above). Opposing regulatory processes for intact proteins and their proteolytic fragments or for proteins carrying different posttranslational modifications may explain why a few proteins were upregulated in one spot (e.g., intact protein) and downregulated in another spot (e.g., proteolytic fragment), because these protein pairs always differed in molecular mass.

#### Clone- and stress-specificity of the proteomic responses

**Fig. 12:**
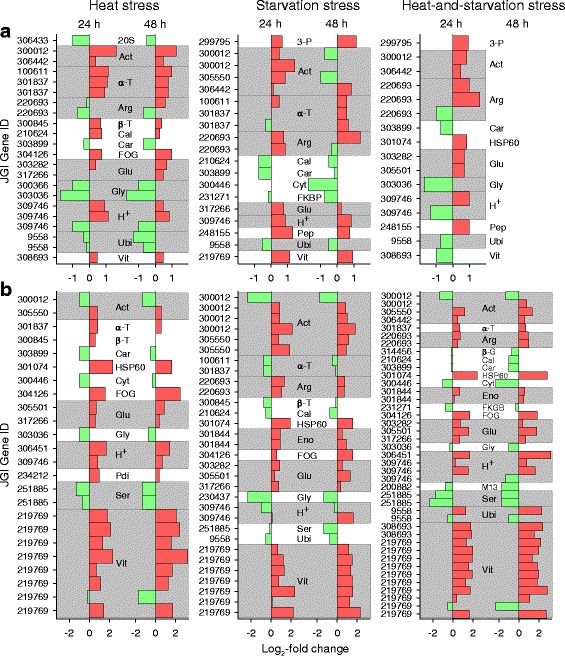
Changes in protein expression in the *D. pulex* clones G and M under heat, starvation, and heat-and-starvation stress. The graphs show log_2_-fold changes in protein expression (stress vs. control conditions) of upregulated (red bars) or downregulated (green bars) proteins (marked by the corresponding gene ID; in full: JGI_V11_gene ID) in the *D. pulex* clones (a) G and (b) M after 24 h or 48 h of heat, starvation, and heat-and starvation stress (data from Tables 3, 4, and 5). Gray background colors indicate the occurrence of more than one protein with identical function

The number of DEPs decreased in animals of the less heat tolerant clone G (22, 19, and 16 proteins) (Fig. 12a) and increased in animals of the more heat tolerant clone M (24, 31, and 41 proteins) (Fig. 12b) in the order heat, starvation, and heat-and-starvation stress, and their number was always higher in clone M than in clone G under each type of stress. Moreover, the mean intensities of stress-induced expression changes (mean log_2_-fold changes) were almost always more negative or more positive in clone M than in clone G (Table 6), and the greatest log_2_-fold changes were only − 1.74 and 1.63 in clone G but − 2.32 and 3.21 in clone M.

**Table 6:** Mean changes in protein expression in the *D. pulex* clones G and M under stress

	Clone G				Clone M			
	Downregulation		Upregulation		Downregulation		Upregulation	
	48 h	24 h	24 h	48 h	48 h	24 h	24 h	48 h
Heat	−0.93	−0.63	0.85	0.70	−0.96	−0.84	1.41	1.62
Starvation	−0.65	−0.47	0.76	0.77	−0.68	−0.85	1.24	1.26
Heat-and-starvation	–	−1.05	0.94	–	−1.24	−0.68	1.31	1.77

Mean log_2_-fold changes (stress vs. control conditions) of down- or upregulated proteins in the *D. pulex* clones G and M after 24 h or 48 h of heat, starvation, and heat-and-starvation stress (data from Fig. 12)

There were also qualitative differences in protein expression between animals of the *D. pulex* clones G and M. Clone M showed strong and significant upregulations of a vitellogenin-SOD fusion protein and a heat shock protein (HSP60) under each type of stress (Tables 3, 4, and 5). In clone G, these proteins were upregulated only under starvation stress (vitellogenin-SOD) and heat-and-starvation stress (HSP60) (Tables 4 and 5). A leucine-rich repeat (LRR) protein (FOG, leucine-rich repeat, with the acronym FOG standing for Fuzzy Orthologous Groups [23]), which is likely involved in the formation of protein-protein interactions [24], was consistently upregulated in clone M but only under heat stress in clone G. However, there were also common principles of protein regulation under stress (Fig. 12). Enzymes involved in protein (carboxypeptidase A2, serine endopeptidase, M13 family peptidase), carbohydrate (glycoside hydrolases, β-glucosidase), and lipid metabolism (cytosolic fatty acid binding protein) were frequently downregulated under stress, excluding only 3-phosphoglycerate kinase, peptidase S1, and enolase. An ubiquitin/ubiquitin-like protein was often downregulated, and actins, an arginine kinase, glutathione transferases, H^+^-transporting ATPases, and tubulins were frequently upregulated in both clones.

**Fig. 13:**
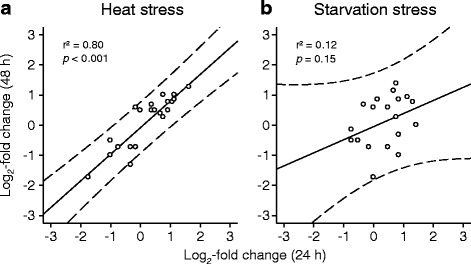
Exemplary correlations between the changes in protein expression under two stress conditions. Linear regression analysis (straight line; dashed curves: 95% prediction intervals) of the log_2_-fold changes in protein expression (stress vs. control conditions) of proteins with identical spot and gene ID from the *D. pulex* clone G after 24 h (x-axis) and 48 h (y-axis) of (**a**) heat or (**b**) starvation stress provided r^2^ and *P* values specifying, in this case, the dependency of protein expression after 48 h of stress on that after 24 h of stress. The r^2^ and *P* values of all correlations performed are shown in Table 7

Correlation analyses of the expression changes of proteins with identical spot and gene ID showed in clone G a tight correlation between the log_2_-fold changes after 24 h and 48 h of heat stress (Fig. 13a) but no correlation between the log_2_-fold changes after 24 h and 48 h of starvation stress (Fig. 13b). Further stress- and clone-specific correlation analyses (Table 7) revealed in animals of clone G additional correlations only between the log_2_-fold changes after 24 h of heat or starvation stress and those after 24 h of heat-and-starvation stress. In animals of clone M, however, there were always significant correlations between the log_2_-fold changes after 24 h and 48 h, after the 24-h periods, or after the 48-h periods of any type of stress. Thus, the stress-induced changes in protein expression were highly similar in animals of clone M. Contrasting the *D. pulex* clones G and M revealed only one significant correlation between the log_2_-fold changes after 24 h of starvation stress.

**Table 7:** Statistical data from linear regression analyses

Clone G										
	48 h heat		24 h starvation		48 h starvation		24 h heat-and-starvation			
	r^2^	*P*	r^2^	*P*	r^2^	*P*	r^2^	*P*		
24 h heat	0.80	< 0.001	0.00	0.97			0.87	< 0.001		
48 h heat					0.42	0.06				
24 h starvation					0.12	0.15	0.90	0.001		
Clone M										
	48 h heat		24 h starvation		48 h starvation		24 h heat-and-starvation		48 h heat-and-starvation	
	r^2^	*P*	r^2^	*P*	r^2^	*P*	r^2^	*P*	r^2^	*P*
24 h heat	0.81	< 0.001	0.48	0.004			0.81	< 0.001		
48 h heat					0.68	< 0.001			0.75	< 0.001
24 h starvation					0.70	< 0.001	0.37	0.004		
48 h starvation									0.84	< 0.001
24 h heat-and-starvation									0.82	< 0.001
Clone G versus clone M										
	24 h heat		48 h heat		24 h starvation		48 h starvation		24 h heat-and-starvation	
	r^2^	*P*	r^2^	*P*	r^2^	*P*	r^2^	*P*	r^2^	*P*
24 h heat	0.15	0.39								
48 h heat			0.14	0.41						
24 h starvation					0.60	0.008				
48 h starvation							0.00	0.86		
24 h heat-and-starvation									0.22	0.13

r^2^ and *P* values from linear regression analyses (see Fig. 13), which specify the degree of correlation between the expression changes (log_2_-fold changes) of proteins with identical spot and gene ID in the case of two stress conditions and one clone (top, *D. pulex* clone G; center, *D. pulex* clone M) or in the case of two clones (clone G vs. clone M) and one stress condition (bottom)

**Fig. 14:**
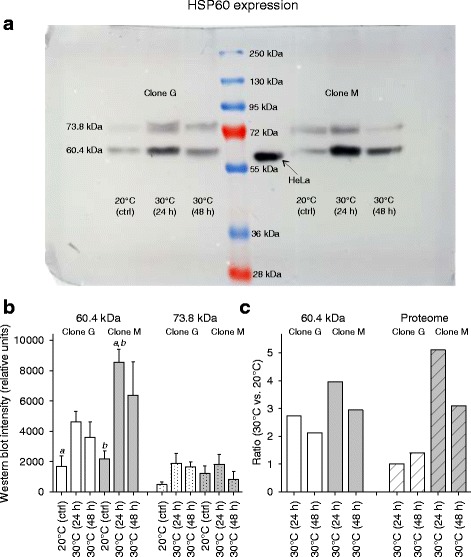
Expression of the heat shock protein HSP60 in the *D. pulex* clones G and M under heat stress. Expression of the heat shock protein HSP60 was determined in the *D. pulex* clones G and M under heat stress by western blot analyses using control animals (20 °C, ad libitum feeding) and animals exposed to 24 h or 48 h of heat stress (30 °C, ad libitum feeding). **a** Representative gel showing for clone G (left) and clone M (right) intensity changes of two HSP60 bands (at 73.8 and 60.4 kDa) under control (ctrl) and heat stress conditions (24 h, 48 h), with the protein marker at the center and heat-shocked HeLa cells as positive controls. **b** Mean intensities (*Daphnia* HSP60 vs. HeLa HSP60) ± standard deviation (*n* = 3 experiments per clone and condition) of the 60.4 kDa or 73.8 kDa (dotted filling pattern) bands from clone G (white bars) or clone M (gray bars) under control or stress conditions. c Ratios (i.e., expression changes of HSP60 in the contrast 30 °C vs. 20 °C) from western blot analysis (60.4 kDa HSP 60) or from proteomics (diagonal filling pattern; see Figs. 10, 11) for clone G (white bars) or M (gray bars). Small letters indicate significant differences between animals of clone M after 24 h of heat stress and control animals of both clones (*a*, *b*; *P* < 0.05, one-way anova and Student-Newman-Keuls multiple comparison procedure)

To verify the proteomic results, the heat-induced expression changes (24 and 48 h at 30 °C) of a prominent protein (JGI gene ID: 301074, Chaperonin ATPase, Cpn60/Hsp60p; e.g., Table 3) were checked in the *D. pulex* clones G and M by western blot analysis. Two distinct bands were found at 73.8 and 60.4 kDa (Fig. 14a). The molecular mass of the 60.4 kDa HSP60 matched quite well its predicted molecular mass (M_r_*p* = 61.4 kDa; Table 3), and it showed markedly higher expression levels than the 73.8 kDa HSP60 (Fig. 14b). Only animals of clone M showed a significantly higher 60.4 kDa HSP60 level after 24 h of heat stress in comparison to the control animals of both clones. The ratios between the 60.4 kDa HSP60 levels at 30 °C and 20 °C were also higher in clone M than in clone G (Fig. 14c).

##### Congruent sets of transcripts and proteins

**Fig. 15:**
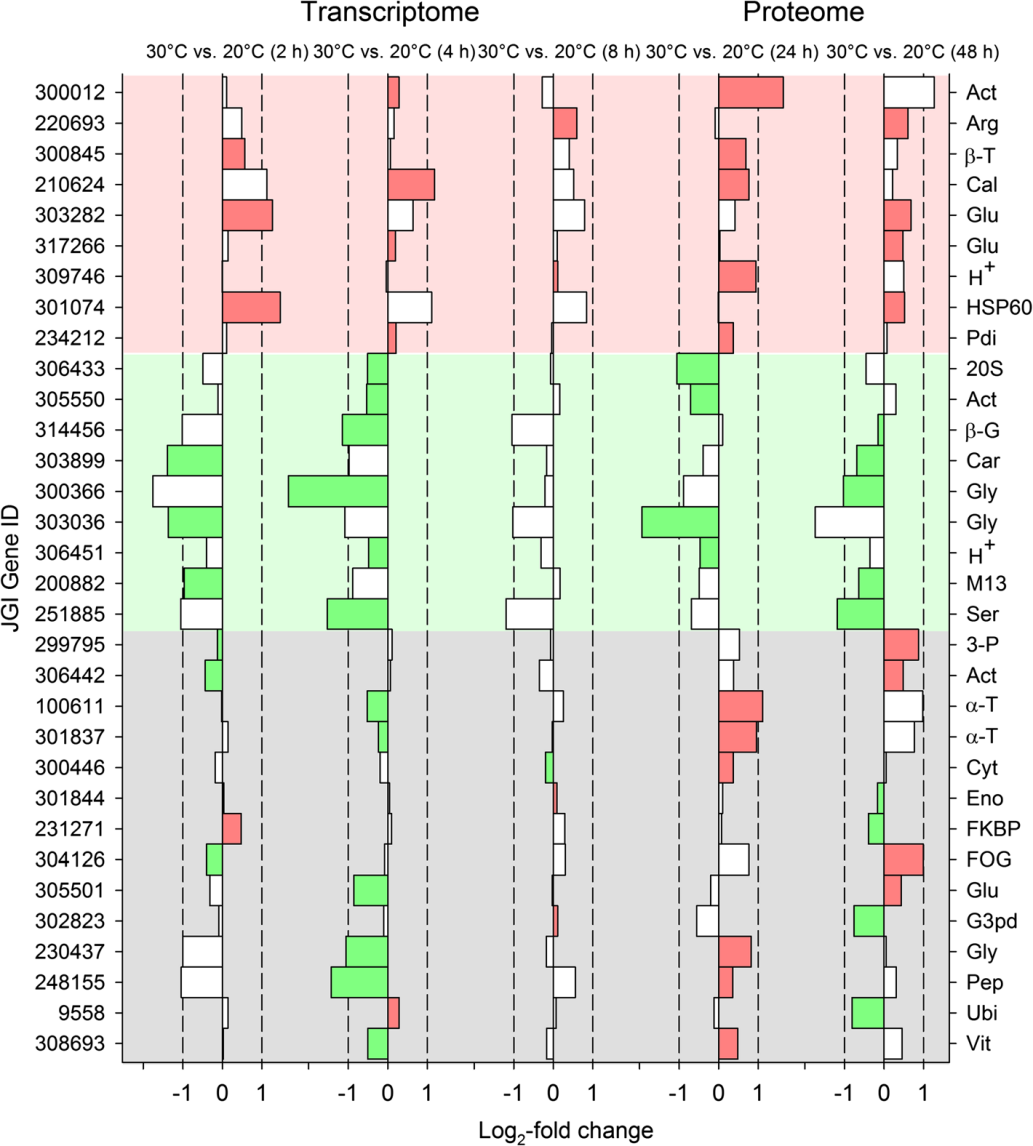
Temporal changes in mRNA and protein levels in clone G under acute heat stress. The transcriptomic and proteomic data from the *D. pulex* clone G under acute heat stress allowed the comparison of temporal changes in the mRNA and protein levels (log_2_-fold changes between stress and control conditions) from identical genes (identical gene IDs). In 18 cases, the mRNA and protein levels showed the same regulatory direction (positive, top; negative, center), whereas antagonistic regulation was observed in 14 cases (bottom). Red, green, and gray background colors separate upregulated, downregulated, and antagonistically regulated genes. Red and green bars mark the most positive or most negative expression changes. Dashed vertical lines indicate log_2_-fold changes of − 1 and 1

The availability of transcriptomic and proteomic data for animals of clone G under acute heat stress suggested a comparative analysis of the temporal courses in the mRNA and protein levels from identical genes. Such a comparison, however, has some limitations as the times of heat exposure (maximally 8 h in case of transcriptomics; maximally 48 h in case of proteomics) and the times of measurement (2, 4, and 8 h in case of transcriptomics; 24 and 48 h in case of proteomics) were quite different. Performing nonetheless such an analysis by sorting the transcripts and proteins from identical genes according to whether the greatest changes in mRNA and protein level were both positive or both negative resulted in 18 agonistic regulations (Fig. 15). Antagonistic regulations were found in 14 cases. Actin, arginine kinase, β-tubulin, calreticulin, two glutathione transferases, an H^+^-transporting ATPase, HSP60, and protein disulfide isomerase were positively regulated on mRNA and protein levels, and a 20S proteasome element, another actin, β-glucosidase, carboxypeptidase, two glycoside hydrolases, another H^+^-transporting ATPase, a M13 family peptidase, and a serine endopeptidase were negatively regulated. The antagonistically regulated set included another actin, two α-tubulins, and seven proteins with quite different functions (3-phosphoglycerate kinase, cytosolic fatty-acid binding protein, the LRR protein (FOG, leucine-rich repeat), glutathione transferase, glycoside hydrolase, peptidase S1, and vitellogenin) in the case of upregulated protein expression and another four functionally different proteins (enolase, FKBP-type peptidyl-prolyl cis-trans isomerase, glyceraldehyde 3-phosphate dehydrogenase, and ubiquitin) in the case of downregulated protein expression.

## Discussion

### Transcriptomic responses of the *D. pulex* clone G to acute heat stress and temperature acclimation

The transcriptomic response to acute heat stress comprised many hundred DEGs. Upregulations dominated over downregulations after 2 h of stress, whereas the opposite was true after 4 and 8 h of stress (Figs. 1a-c, 2c). Many upregulated KOG-identified DEGs (Fig. 4) encode stress proteins (e.g., molecular chaperones) (Fig. 5a), and the downregulated KOG-identified DEGs included genes for protein and carbohydrate metabolism (Fig. 5b, c). Thus, the gene expression pattern under acute heat stress involved an early expression of stress genes, followed by a downregulated expression of metabolic genes (see Fig. 8b), which likely reflects a transcriptional switch from routine products of gene expression (e.g., hydrolases) to the sudden need for specific products (stress proteins).

The transcriptomic response to temperature acclimation differed according to the temperature level. A few DEGs were mostly upregulated in a medium-temperature range (20 °C vs. 10 °C acclimation), whereas much more DEGs were predominantly downregulated in a high-temperature range (24 °C vs. 20 °C acclimation) (Figs. 1d-e, 2c). The low number of identical DEGs between these two contrasts (Figs. 2b, 8c) additionally indicates different principles of acclimation. Systemic adjustments are feasible for *Daphnia* in the medium-temperature range but difficult in the high-temperature range. In the medium-temperature range, adjustments in the flow rate of the ventral water current (ventilatory flow rate), from which *Daphnia* extracts oxygen and plankton [19], as well as in the perfusion rate (circulatory flow rate) allow flexible responses to changes in temperature (or oxygen, nutrient, and energy supply and demand), which may be supported by upregulated gene expression. In the high-temperature range, where ventilation and perfusion rates approach their maximum levels [19], additional or other measures must be taken, which obviously are based on a changed gene expression pattern, to eventually match energy supply and demand.

The upregulated DEGs in the contrast 20 °C vs. 10 °C acclimation (Fig. 6a) included genes for trypsin and carbohydrases. The upregulated DEGs in the contrast 24 °C vs. 20 °C acclimation (Fig. 6b) also included genes for trypsins (Fig. 7c) and carbohydrate metabolism and transport (Fig. 7d). Not only the upregulated DEGs for carbohydrases but also the upregulated DEGs for permeases may be related to digestive processes because permeases are involved in passive or secondary active transports of dissolved substrates over cell membranes (epithelial uptake of monomers). The upregulated DEGs for UDP-glucuronosyl/−glucosyl transferases may participate in the removal of metabolic waste products. These results suggest an upregulated gene expression for the digestion of proteins and carbohydrates to match the higher metabolic demands at higher temperatures [25]. Many downregulated DEGs in the contrast 24 °C vs. 20 °C acclimation encode proteins for translational processes (e.g., ribosomal proteins) (Fig. 7a) and collagens (Fig. 7b), which indicates decreasing investments in protein biosynthesis, cuticle construction, and growth at an acclimation temperature (24 °C), which is only a few degrees celsius below the long-term temperature limit of these animals in our laboratory. Thus, the acclimation temperature of 24 °C evidently requires a gene expression pattern that causes an increase in energy intake (via digestion) and a decrease in energy expenditures.

Overall principles of gene expression under chronic temperature changes were not detectable because only 26 DEGs with quite diverse functions were shared by the contrasts 20 °C vs. 10 °C and 24 °C vs. 20 °C (Figs. 2b, 8c). The gene for a β-transducin repeat-containing protein (β-TrCP; JGI gene ID: 304793), however, may be interesting because it was highly upregulated under acute heat stress as well (Fig. 9). β-TrCP has been reported to play important roles in regulating cell cycle checkpoints (for instance, in response to DNA damage) and promoting protein translation and cell growth [26, 27]. Overall principles of gene expression under acute and chronic temperature changes were also not detectable (Fig. 3), with the exception of the upregulated gene for β-TrCP (Fig. 9), which was likely due to the big differences in temperature (30 °C vs. 24 °C) and exposure time (8 h vs. 12 weeks and more) between stress and acclimation conditions. As discussed above, the transcriptomic response to acute heat stress involved an early expression of stress genes (Fig. 5a), followed by a downregulated expression of metabolic genes (Fig. 5b, c), whereas the transcriptomic response in the high-temperature range of acclimation included a downregulation of genes for anabolic processes (Fig. 7a, b) and an upregulated expression of metabolic genes (Fig. 7c, d). The 13 identical DEGs between acute heat stress and 20 °C vs. 10 °C acclimation (Fig. 9a) included genes for amino acid or carbohydrate metabolism and transport as well as for proteins that are possibly involved in the removal of waste materials and foreign substances (carboxylesterases, peroxidase/oxygenase, plasma membrane glycoproteins CD36, UDP-glucuronosyl and UDP-glucosyl transferase) [28, 29]. The 34 identical DEGs between acute heat stress and 24 °C vs. 20 °C acclimation (Fig. 9b) included genes for amino acid or carbohydrate metabolism and transport, for four proteins possibly involved in the removal of waste materials and foreign substances (see above), and for five proteins likely involved in stress responses (caspase, ER-associated oxidoreductin, glutathione S-transferase, serine/threonine protein kinase and endoribonuklease (sensor of the unfolded protein response, UPR), and thioredoxin/protein disulfide isomerase) [30, 31].

### Proteomic responses of the *D. pulex* clones G and M to acute heat, starvation, and heat-and-starvation stress

The resistance to heat or heat-and-starvation stress was much lower in clone G than in clone M (Table 1) and although animals of both clones were equally impaired by starvation stress, the protein concentration decreased only in animals of clone G (Table 2), which implies reduced protein biosynthesis and/or enhanced autophagic proteolysis in clone G but not in clone M.

Contrasting the proteomes of the *D. pulex* clones G and M under heat, starvation, and heat-and-starvation stress showed distinct differences in protein expression patterns. The number of DEPs was much higher in clone M (Fig. 12b) than in clone G (Fig. 12a) at each type of stress. Expression changes were also higher, and upregulations dominated over downregulations in clone M, whereas they were more or less balanced in clone G (Table 6). The number of DEPs increased in clone M and decreased in clone G in the order heat stress, starvation stress, and heat-and-starvation stress (Fig. 12). The expression changes of identical DEPs were similar over time (24 and 48 h) and, at one time point, relatively uniform between different types of stress in clone M but not in clone G (Table 7). The significantly stronger proteomic responses of clone M to any type of stress in comparison to those of clone G indicate a much higher energy availability. The low number of DEPs in clone G under heat-and-starvation stress suggests an impairment of translational processes resulting in a reduced survival rate, whereas the high number of DEPs in clone M under this stress condition reflects intensified translational processes (e.g., expression of stress proteins) improving the survival rate of this clone. The similarities in the proteomic responses of clone M over time and to different types of stress are probably related to constant requirements under any type of stress, including particularly an active preservation of the energy balance. The rather passive reactions of clone G under stress included, for instance, reduced protein biosynthesis and/or enhanced autophagic proteolysis under starvation (see above).

Particularly remarkable in the more heat tolerant clone M were strong expression changes for an HSP60 and for a vitellogenin (VTG) fused with a superoxide dismutase (SOD) domain (VTG-SOD). The proteomic analyses showed an upregulation of the HSP60 protein in clone M under any type of stress but only under heat-and-starvation stress in clone G (Fig. 12). Western blot analyses also showed a much higher upregulation of this HSP60 in clone M than in clone G under heat stress (Fig. 14). HSP60 induction is part of the cellular stress response [32, 33]. Mitochondrial HSP60 supports protein import and folding [34] and DNA metabolism [35], and cytoplasmic HSP60 counteracts apoptosis [36]. The function of HSP60 under heat stress is presumably related to protein repair, whereas transmembrane transports of proteins and/or DNA metabolism are potential key functions of HSP60 under starvation stress. Other heat shock proteins were likely also upregulated (see Fig. 5a) but were not detected in the present proteomic study. The synthesis of heat shock proteins requires, as any protein biosynthesis, a large amount of ATP [37]. The lower stress-induced expression of HSP60 (and many other proteins; see above) in clone G suggest substantial differences in ATP availability between both clones, which may be related to a higher level of stress-induced cellular damage in clone G which negatively affects ATP production.

This hypothesis is supported by the clear differences in VTG expression between both clones. There were two types of upregulated VTGs, with the VTG-SOD variant predominantly upregulated in clone M (Tables 3, 4, and 5). VTGs are precursors of the yolk protein vitellin, which is a lipoglycoprotein employed as a vehicle to supply developing embryos with carbohydrates, lipids, proteins, and other essential resources [38]. SOD is a major ROS-scavenging enzyme, which converts superoxide into the less harmful hydrogen peroxide, which is then degraded by catalase [39]. To date, a VTG-SOD has only been reported for two other crustaceans, *Daphnia magna* [40] and *Artemia parthenogenetica* [41]. *Daphnia* or *Artemia* release ephippia or diapause cysts (encysted embryos) to withstand environmental stress, and it has been suggested that the SOD domain of these VTGs is important during the development of these resting stages by enhancing stress tolerance [41]. The upregulation of VTG-SOD, especially in clone M, indicates the production of ephippia as an emergency response to stress, whereas a lack of ATP possibly has prevented clone G from investing in adequate reproductive strategies under stress. VTG-SOD was upregulated in the heat-sensitive clone G only under starvation stress (Table 4), which may have been supported by an autophagic degradation of internal proteins under this stress condition (Table 2).

Despite the differences between both clones, there were also shared principles of protein regulation. Actins, arginine kinase, glutathione transferases, H^+^-transporting ATPases, and tubulins were frequently upregulated (Fig. 12). The upregulation of actins and tubulins may serve to counteract stress-induced impairments of the cytoskeleton. Arginine kinase is a phosphotransferase that plays a key role in cellular energy metabolism by catalyzing the production of phosphagens (phosphoarginine), from which ATP can be rapidly replenished [42]. The upregulation of glutathione transferases may reflect a higher production of stress-damaged cell components and molecules, which can be exported, after glutathionylation, by membrane transporters (e.g., ABC transporters) [43]. H^+^-transporting ATPases can energize apical plasma membranes by generating potential differences via the outward transports of protons [44], in this way facilitating membrane transports. Proteins that were frequently downregulated under any type of stress included enzymes involved in protein, carbohydrate, and lipid metabolism (Fig. 12), which was also characteristic of the transcriptomic response of clone G under acute heat stress.

### Links between mRNA and protein level

The common set of heat-induced mRNAs and proteins in clone G comprised 32 elements (Fig. 15). The positively regulated set consisted of five stress proteins (calreticulin, two glutathione transferases, HSP60, protein disulfide isomerase), two cytoskeletal proteins (actin, β-tubulin), arginine kinase, and an H^+^-transporting ATPase. Calreticulin prevents misfolded proteins in the ER from proceeding to the Golgi apparatus, which finally causes their degradation. It is also involved in the regulation of Ca^2+^ homeostasis [45]. In addition, its mRNA is expressed under acute cellular stress [46], with calreticulin then interacting with other molecular chaperones during the recovery from acute stress [47]. Calreticulin accumulation and function are also linked to apoptotic processes [48, 49]. Protein disulfide isomerases were also reported to be involved in apoptotic processes [49], and they support protein folding within the ER by catalyzing thiol-disulfide exchanges (formation of disulfide bonds between cysteine residues) [50]. The negatively regulated set comprised three proteases (carboxypeptidase, peptidase S1, serine endopeptidase), three carbohydrases (β-glucosidase, two glycoside hydrolases), a 20S proteasome element and another actin and H^+^-transporting ATPase. The antagonistically regulated set included, in the case of upregulated protein expression, three cytoskeletal proteins (two α-tubulins and another actin) and seven proteins with quite different functions and, in the case of downregulated protein expression, another four functionally different proteins (Fig. 15). However, it should also be noted that the applied criterion to assort the transcripts and proteins (see Results) may not always have correctly captured the regulatory direction as the time resolution of these measurements was rather limited. In case of the LRR protein (FOG, leucine-rich repeat; JGI gene ID: 304126), for instance, the increasing mRNA level after 8 h of heat stress might have triggered the increasing protein level after 48 h of heat stress. In general, mRNA levels are very dynamic quantities, and even protein levels may change rapidly, which requires in principle (but hardly feasible in practice) a high time resolution of measurements to precisely correlate changes in mRNA and protein levels [18]. Nonetheless, the proteomic data presented herein show, despite a relatively low number of common mRNA and protein species, similar results to the transcriptomic study, demonstrating an upregulation of stress-related genes and a downregulation of proteases and carbohydrases upon acute heat stress (see above).

## Conclusions

The transcriptomic and proteomic responses of the differently heat tolerant clones G and M to environmental changes comprised environment-specific and clone-specific elements. Thus, different time courses (acute or chronic changes in temperature) or intensities (medium or high acclimation temperatures) of the environmental factor temperature evoked clearly different transcriptomic responses in clone G. Defining features of the transcriptomic response to acute heat stress were an early expression of stress genes, followed by a downregulated expression of metabolic genes (e.g., hydrolases), which likely reflects a transcriptional switch from routine products of gene expression to the sudden need for stress proteins. The effectiveness of systemic adjustments (e.g., ventilation, perfusion) at acclimation temperatures between 10 °C and 20 °C lowered the need for gene expression, whereas acclimation temperatures between 20 °C and 24 °C, which push the systemic adjustment possibilities of these animals to their limits, primarily required molecular adjustments. Differential gene expression in the contrast 24 °C vs. 20 °C included a downregulated expression of genes for anabolic processes (protein biosynthesis and growth) and an upregulated expression of metabolic genes (hydrolases), which likely reduced the energy expenditures and increased energy generation from feed.

Aside from common principles of protein regulation such as a downregulation of enzymes involved in protein, carbohydrate, and lipid metabolism under stress, different clonal properties strongly affected the protein expression patterns in clone G and M. The stress-induced proteomic responses in the more heat tolerant clone M were superior to those in the less heat tolerant clone G. Clone M showed, in contrast to clone G, higher numbers and expression changes of DEPs, a dominance of upregulated over downregulated protein expression, and an increase in the number of DEPs in the order heat, starvation, and heat-and-starvation stress. The adaptation of clone M to its more stressful habitat (i.e., a pond with strong temperature fluctuations) was likely responsible for its higher capacity for stress protein expression (e.g., HSP60). An improved protection and repair of macromolecules (including the energy supply mechanisms) in clone M provide the preconditions for an unimpaired or even rising energy availability under heat or heat-and-starvation stress, which then allowed intensified translational processes and other appropriate measures for the survival of this clone, including VTG-SOD expression and the initiation of resting egg production as an emergency reaction. The strong similarities in the proteomic responses of clone M over time (24 and 48 h) and to all types of stress are probably due to constant requirements, which include particularly a preservation of the energy balance to counteract the adverse effects of heat or starvation (see Introduction). Macromolecular damage and impaired energy availability were likely the reasons for the weaker proteomic responses and the lower survival rates of clone G under heat stress and particularly heat-and-starvation stress. Only under the less harmful starvation stress, clone G was able to upregulate VTG-SOD expression, which, however, was at the expense of many other protein biosyntheses and/or led to autophagic processes as the internal protein stores of this clone decreased severely.

## Methods

### Animals

The *Daphnia pulex* clones G and M were characterized by allozyme analysis [51] using eight different enzymes, resulting in differences in three cases (aldehyde oxidase, lactate dehydrogenase, malate dehydrogenase). Clone G was isolated from a flooded and eutrophic quarry at Gräfenhain near Dresden, Germany (N50°49′04″, E10°42′02″) in 2002. This wind-sheltered, monomictic quarry with a surface area of 440 m^2^ and a mean depth of 7 m is characterized by strong temperature stratification [52]. Clone M was isolated from a highly eutrophic seasonal pond in Münster, Germany (N51°57′48″, E7°34′38″) in 2007. In this temporarily flooded pond with a maximal water depth of 1 m, strong fluctuations in water level and temperature occur.

Experimental animals were kept in 1.5 L of M4 medium [53] at three different temperatures (i.e., 10 °C ± 0.5 °C, 20 °C ± 0.3 °C, and 24 °C ± 0.3 °C) under normoxia (*P*o_2_ ≥ 20 kPa) and a 16:8 h light:dark photoperiod for at least twelve weeks. The animals were daily fed ad libitum (2.5 mg C/L) with *Desmodesmus subspicatus* (SAG 53.80, Göttingen, Germany). To maintain parthenogenetic reproduction, three-quarters of the medium were exchanged twice a week, and animals were kept at a density of 50-100 individuals per beaker. Any males or ephippial females were removed on appearance. Only adult female animals with a body length between 2 and 2.5 mm (between the base of the apical spine and the anterior part of the head) and carrying parthenogenetic eggs and embryos were used for experiments.

Transcriptomic studies were carried out only on animals of clone G, for which the following experimental conditions applied (Additional file 7: Figure S6). Time-resolved experiments (acute heat stress) were conducted on long-term 20 °C-acclimated animals (see above), which were exposed for three different time periods (2, 4, and 8 h) to either 30 °C ± 0.2 °C (test conditions) or 20 °C ± 0.3 °C (control conditions). These experiments started with the rapid transfer of 50 animals each, which were collected in a sieve (mesh size: 1.2 mm), from the medium at 20 °C to a set of beakers containing the same medium at 30 °C. As control, 50 animals each were transferred from the medium at 20 °C to another set of beakers with the medium temperature at 20 °C. Animals were not fed during these experiments; but all animals were in good physical condition during and after the different heat exposures. Chronic temperature experiments were conducted on long-term 10 °C-, 20 °C-, and 24 °C-acclimated animals (see above). Immediately after heat exposure or temperature acclimation, animals were transferred to 1.5-mL microcentrifuge tubes and shock-frozen in liquid nitrogen after removing adhering water with a tissue paper. Samples were short-term stored at − 80 °C. For all experimental conditions, four independent replicates (50 animals each) were used.

Proteomic studies were carried out on animals of the *D. pulex* clones G and M, for which the following experimental conditions applied (Additional file 7: Figure S6). For each experiment, 25-30 long-term 20 °C-animals (see above) were either used as control animals or transferred for 24 h or 48 h to pre-tempered media of different temperature and food supply: 30 °C, ad libitum feeding (*D. subspicatus*) (heat stress); starvation, 20 °C (starvation stress); 30 °C, starvation (heat-and-starvation stress). In case of experiments with fed animals, ad libitum food supply was stopped 12 h before sampling, with the animals then kept in algae-free pre-tempered M4 medium, to minimize contributions by algae proteins. The media were always mildly aerated using a membrane pump. Immediately after 24 h or 48 h of incubation, regularly swimming animals were collected by sieving and after gently drying them with a paper tissue to remove adhering water, their fresh weight was determined, before they were shock-frozen in liquid nitrogen.

### Transcriptomics

#### Microarray analysis

The microarray platform consisted of a 12-plex 60 nt-oligonucleotide *D. pulex* microarray, which is described elsewhere [20]. For each treatment, four independent RNA samples were processed and the labeled cDNA were competitively hybridized on four replicate microarrays including dye swaps (Additional file 8: Table S2). Analysis of the gene expression profiles of long-term-acclimated *D. pulex* comprised direct comparisons of all acclimation conditions (log_2_-transformed expression ratios between 20 °C and 10 °C, 24 °C and 20 °C as well as 24 °C and 10 °C). For the time-resolved experiments, log_2_-transformed expression ratios were calculated between 30 °C and 20 °C exposures at different time points (2, 4, and 8 h after transferring 20 °C-acclimated animals to 30 °C or 20 °C).

#### Sample preparation

Samples for microarray analysis were prepared as described elsewhere [54]. Briefly, total RNA was extracted from *D. pulex* with the RNeasy Kit (Qiagen, Hilden, Germany) and DNA contamination was removed by DNase treatment (Qiagen) following the manufacturer’s protocol. RNA quantity and quality was determined by use of spectrophotometry (NanoDrop 1000; Thermo Scientific, Wilmington, NC, USA) and Bioanalyzer 2100 technology (Agilent, Santa Clara, CA, USA), respectively. Samples were short-term stabilized in RNAstable as specified by the manufacturer (Biomatrica, San Diego, CA, USA). To obtain adequate amounts of RNA from all biological samples one microgram of total RNA was linear-amplified with the Message Amp II aRNA Amplification Kit (Ambion, Carlsbad, CA, USA) following the manufacturer’s instructions. Quantity and quality of amplified RNA (aRNA) were determined as described above. Double-stranded (ds) cDNA was synthetized from aRNA samples with SuperScript double-stranded cDNA Synthesis Kit (Invitrogen, Carlsbad, CA, USA). Following RNase treatment (Promega, Madison, WI, USA) and clean up, ds cDNA samples were labeled using randomly either Cy3- or Cy5-coupled nonamer primers (NimbleGen Dual-Color Labeling Kit, Roche NimbleGen, Madison, WI, USA). Quantity and quality of labeled samples were again determined as described earlier. Subsequently, labeled samples were pooled according to the experimental design (Additional file 8: Table S2), and hybridization to respective arrays was accomplished with the NimbleGen Hybridization Kit (Roche Nimblegen). After hybridization, microarray glass slides were washed, dried and stored in the dark until fluorescence was measured. Finally, array slides were scanned (NimbleGen MS 200 Microarray Scanner; Roche NimbleGen), and images extracted with NimbleScan v2.6 Software (Roche NimbleGen).

#### Data and statistical analyses

To obtain differential gene expression profiles, data were analyzed as described elsewhere [20]. Briefly, for normalization and analysis, raw data were imported into the statistical software packages R [55] and Bioconductor [56]. All probes were quantile-normalized across chips, samples, and replicates, and differential gene expression levels were obtained from the median log_2_-fluorescence intensity of probes by LIMMA (Linear Models for Microarray Data) functions [57] and t statistics from the empirical Bayes method [58]. Genes printed on the array and analyzed in this study refer to the *Daphnia pulex* genome assembly v1.1 [59]. Significance levels of differential gene expressions were adjusted for multiple testing by calculating the false discovery rate (FDR) according to [60]. Differential gene expression was considered as significant with a 5% FDR cut-off (Q-value ≤ 0.05).

In order to visualize the dissimilarity of gene expression profiles resulting from different temperature treatments, we used a non-metric multidimensional scaling (MDS) analysis. For this purpose, the Euclidian distance between all samples was scaled to three dimensions, with normalized intensities as input and data normalized to either 20 °C acclimation or 20 °C controls (acute heat stress).

All predicted protein-coding gene models were functionally assigned to orthologous groups (euKaryotic Orthologous Groups, KOG) [61] as defined by the Joint Genome Institute [62]. Chi-square tests (*P* ≤ 0.05) were applied to assess enrichment of up- or downregulated DEGs with KOG ID (KOGs) in the different KOG categories (i.e., disproportionate high numbers of up- or downregulated KOGs). The reference set for chi-square testing comprised all KOGs of a specific contrast. Statistical analyses were carried out using R [63], Excel (Microsoft), and SigmaPlot 11.0 (Systat Software, Erkrath, Germany).

### Proteomics and concomitant examinations

#### Survival assay and its statistical evaluation

To test for the survival times of the *D. pulex* clones G and M under different conditions, ten 20 °C-acclimated animals were transferred to pre-tempered media of different temperature and food supply: control conditions, heat stress, starvation stress, and heat-and-starvation stress (see above). The media were mildly aerated using a membrane pump. Any freshly hatched juvenile *Daphnia* were removed. The number of swimming *Daphnia* was counted, and dead (immobile) animals were removed. Results were derived from Kaplan-Meier survival curves, with the statistical significance of differences determined using the Gehan-Breslow test (SigmaPlot 11.0; Systat, Erkrath, Germany).

#### Protein extraction

For the extraction of total soluble protein, rehydration buffer (8 mol/L urea, 2 mol/L thiourea, 4% (*w*/*v*) CHAPS (3-[(3-Cholamidopropyl)dimethylammonio]-1-propanesulfonate), 65 mmol/L DTT (dithiothreitol), 0.5% (*v*/v) ampholyte-containing IPG (immobilized pH gradient) buffer pH 4-7 (Bio-Rad, München, Germany), and one tablet of Complete Mini Protease Inhibitor Cocktail (Roche, Mannheim, Germany) per 3.5 mL of solution) was added to each sample (1:10 w/v), which was then homogenized on ice using a Teflon^®^ pistil. After centrifugation (17,900×*g*, 4 min, 4 °C), the supernatant was mixed 1:2 (*v*/v) with 25% TCA (trichloroacetic acid) and incubated on ice for 70 min. Proteins were precipitated by centrifugation (17,900×*g*, 15 min, 4 °C). The protein pellet was washed ten times by adding ice-cold 80% acetone (containing 0.2% DTT) followed by centrifugations (17,900×*g*, 5 min, 4 °C). The pellet was then resuspended in 110 μL rehydration buffer. Protein concentrations were determined by Bradford assays [64] using a Multimode Reader LB 941 TriStar (filter, F590/10; Berthold Technologies, Bad Wildbad, Germany).

For a validation of proteomic data by Western Blot analyses, aliquots from controls (20 °C) and samples from heat stress experiments (24 h or 48 h at *T* = 30 °C) were kept. Prior to isoelectric focusing (IEF), 350 μg protein was diluted in rehydration buffer to an end volume of 110 μL and washed a second time using the ReadyPrepTM 2-D Cleanup Kit (Bio-Rad) according to the manufacturer’s protocol. The protein was then dried at room temperature for maximally 5 min and resuspended in rehydration buffer to an end concentration of 1 μg/μL. Samples were kept at − 80 °C.

#### Two-dimensional gel electrophoresis

The total protein content of raw extracts of the samples used for later two-dimensional (2D) gel electrophoreses was determined [64]. For isoelectric focusing, linear IPG strips (17 cm, pH 4-7 ReadyStrip™; Bio-Rad) were passively rehydrated for 12 h in 350 μL of rehydration buffer, which additionally contained brom phenole blue. IEF was performed in a manifold tray (IEF-StripholderEttan™IPGphor™ Cup Loading Manifold; Amersham, Piscataway, NJ, USA) on an EttanIPGphor II isoelectric focusing unit (Amersham). 120 μg of protein extract was diluted in 150 μL of rehydration buffer and applied in a cup on the cathodic end. IEF was carried out at 15 °C. Voltage settings comprised 50 V for 5.5 h, a 50-100 V gradient for 1 min, 100 V for 7 h, a 100-1000 V gradient for 10 min, 1000 V for 2 h, a 1000-4000 V gradient for 1 h, 4000 V for 2 h, a 4000-8000 V gradient for 45 min, and 8000 V until 45,000 Vh were reached. Then, IEF Strips were equilibrated for 15 min in 10 mL equilibration buffer (0.05 mol/L Tris (tris(hydroxymethyl)aminomethane), 6 mol/L urea, 30% glycerol, 2% SDS (sodium dodecyl sulfate), pH 8.8), which additionally contained 1% DTT, and another 15 min in 10 mL equilibration buffer, which additionally contained 2.5% iodoacetamide. In the second dimension, protein separation according to molecular mass was carried out using 12% polyacrylamide gels (0.56 mol/L Tris, 0.1% SDS, pH 8.8) in a Protean II xi cell (Bio-Rad). Electrophoresis was performed in a Tris-glycine buffer system [65] at 15 mA for 15 min, and then at 40 mA for 10 h. PageRuler™ Protein Ladder (10-200 kDa; Fermentas, St.-Leon-Rot, Germany) was used for molecular mass calibration. Gels were stained with the fluorescent dye Ruthenium II tris (bathophenanthroline disulfonate) (RuBPs) as described in [66]. Fluorescence intensity due to UV light excitation (UVT-20 M/W; Herolab, Wiesloch, Germany) was documented (Olympus E410, 14-42 mm/ F 3.5-5.6) using Olympus Studio 2 (Olympus, Hamburg, Germany) and analyzed with Delta 2D 4.3 (DECODON, Greifswald, Germany). Gels were warped using the exact warp mode and fusion gels were created using the union mode. Pictures were adjusted with Ulead PhotoImpact X3 (Corel Corporation, Ottawa, Canada).

#### Data and statistical analyses

Preceding the quantification of protein expression from staining intensities in the gels, a normalization procedure was done setting the total spot quantity to 100% for each gel image. Protein expression was assessed by calculating the individual spot quantity in proportion to the total spot quantity for each 2D gel. Spot quantities were calculated in Delta 2D. The statistical significance of changes in protein expression was determined with Student’s t-test (*P* < 0.05) (SigmaPlot 11.0).

#### LC-MS/MS, identification and characterization of proteins

RuBPs-stained protein spots were excised from the gel and subjected to overnight in-gel trypsin digestion according to established protocols [67]. Peptides were resuspended in eluent A (see below) and analyzed by LC-MS/MS (liquid chromatography coupled with tandem mass spectrometry) on an Ultimate 3000 Nanoflow HPLC system (Dionex Corporation, Sunnyvale, CA, USA) coupled to an LTQ Orbitrap XL mass spectrometer (Thermo Finnigan, Bremen, Germany). Eluents were composed of aqueous solutions of 5% (v/v) acetonitrile (JT Baker, Deventer*,* Netherlands)/0.1% (v/v) formic acid (eluent A) and 80% acetonitrile/0.1% formic acid (eluent B). The flow rate was set to 300 nL/min.

The sample (1 μL) was loaded onto a trap column (C18 PepMap100; 300 μm i.d. × 5 mm, 5 μm particle size, 100 Å pore size; Dionex) and desalted using eluent A at 25 μL/min for 4 min. Subsequently, the trap column was switched in series with a capillary column (Atlantis dC18; 75 μm × 150 mm, 3 μm particle size, 100 Å pore size; Dionex), and the following gradients for eluent B were applied for peptide separation: 0-12% (10 min), 12-50% (45 min), 50-100% (2 min), and 100% (5 min). The column was re-equilibrated with 100% eluent A for 10 min. Peptides eluted directly into the nanospray source of the mass spectrometer.

The mass spectrometer was operated in positive ion mode. Survey scans were obtained by FT-MS (Fourier-transform ion cyclotron resonance mass spectrometry) (*m/z* 375-2000) at a resolution of 60.000 FWHM using internal lock mass calibration on *m/z* 445.120025. The five most intense ions were sequentially subjected to CID- (collision-induced dissociation) fragmentation (35% normalized collision energy) in the linear ion trap (MS2). Fragment ions were analyzed in the mass analyzer of the ion trap. Dynamic exclusion was enabled with a repeat duration of 60 s, repeat count of 1 and exclusion mass width of ±5 ppm.

For the identification of peptides, MS2 spectra were matched against the *D. pulex* “Filtered Models v1.1”, the “Frozen Gene Catalog protein database v 1.1” [20] as well as the “allModels of 2007 set” and the *Daphnia*_genes2010 database [68] using the SEQUEST^®^ algorithm implemented in Bioworks 3.3.1 SP1 (Thermo Finnigan), OMSSA 2.1.4 [69], and X!tandem release CYCLONE (2012.10.01; X! Tandem) [70, 71]. Evaluation of the mass spectrometric results, matching and aligning peptides to genes and calculating sequence coverage values were done with Proteomatic [72]. The maximum number of missed cleavages allowed was two. Mass accuracy was set to 5 ppm for MS1 precursor ions and 0.8 Da for product ions. Oxidation of methionine was included as variable parameter. A minimum of three identified peptides and/or sequence coverage of at least 30% were considered necessary for positive protein identification. If multiple proteins in a spot matched these criteria, either the protein with the highest peptide count (NP) or in case of equal peptide counts that with the highest sequence coverage (SC) and the best match between M_r_*e* and M_r_*p* and pI*e* and pI*p* was assigned to the spot (see Table 3 for abbreviations). Putative protein functions were identified by the automated blastp search of the Joint Genome Institute [20]. The derived protein sequences were analyzed for putative N-terminal signal sequences using SignalP V4.0 [73]. M_r_*p* and pI*p* of the mature proteins (proteins without signal peptide) were determined by the ExPASy proteomic tool “Compute pI/MW” [74].

#### HSP60 quantification with western blots

To validate exemplarily results from the proteomic studies, heat shock protein 60 (HSP60) quantities were determined by western blots using the already frozen samples for 2D gel electrophoresis (i.e., protein extracts diluted in rehydration buffer; see above). 3× loading buffer (19 mmol/L Tris, 9% SDS, 30% glycerin, 75 mmol/L DTT, and a spatula tip of bromophenol blue) was added at a ratio of 2:1 between sample and loading buffer. Equal amounts of protein (120 μg protein/lane) were subjected to 10% sodium dodecyl sulfate polyacrylamide electrophoresis (SDS-PAGE) along with the protein mass marker (PageRuler Plus Prestained, 11-250 kDa; Fermentas, St.-Leon-Rot, Germany) and standardized extracts from heat-stressed human HeLa cells (Biotrend, Köln, Germany) as positive control. Separated proteins were blotted (18.5 h, 100 mA, *T* = 20 °C) to a polyvinylidene fluoride membrane (Roti®-PVDF membrane, pore size 0.45 *μ*m; Roth, Karlsruhe, Germany). To validate blotting and verify equal protein amounts in the lanes [18, 75], proteins on the membrane were stained with 0.2% Ponceau S in 1% acetic acid. After blocking the membrane in saline phosphate buffer containing 5% dried milk, the blot was probed using primary anti-HSP60 (SPA-805, dilution 1:1000; Biotrend) as described in [76] and subsequently goat anti-rabbit horseradish peroxidase-conjugated secondary antibody solution (A6154, dilution, 1: 10000; Sigma Aldrich, Germany). Detection of the chemiluminescence upon incubation with luminol (final concentration, 1.25 mmol/L) and p-coumaric acid (final concentration, 0.2 mmol/L) was performed by exposure to a photographic film (Amersham Hyperfilm ECL, GE Healthcare, Germany). For each digitized gel image (Epson Perfection V700 Photo; EPSON, Meerbusch, Germany), HSP60 quantities were normalized with respect to the HSP bands of the HeLa cells extracts. Intensities were evaluated using ImageJ 1.47 m (W. Rasband, National Institutes of Health, USA), and intensity ratios (*Daphnia* HSP60 vs. HeLa HSP60) were calculated. Pictures were adjusted with Ulead PhotoImpact X3.

## Additional files

Additional file 1: **Table S1.** KOG-identified DEGs shared by the contrasts under acute heat stress. (PDF 125 kb)

Additional file 2: **Figure S1.** Two-dimensional protein gels from the 20 °C-acclimated *D. pulex* clones G and M. The RuBPs-stained 2D gels from control animals (20 °C, ad libitum feeding) of (a) the *D. pulex* clone G [fusion image from *n* = 5 gels (biological replicates)] and (b) the *D. pulex* clone M [fusion image from *n* = 4 gels (biological replicates, 25-30 animals each)] served as reference for the excision of protein spots for mass spectrometry (pI: 4.5-6, molecular mass: 20-150 or 20-100 kDa). Red characters are spot identifiers (IDs). (PDF 118 kb)

Additional file 3: **Figure S2.** Two-dimensional protein gels from the *D. pulex* clone G under starvation stress. The 2D gels, which are fusion (averaged) images from a varying number (*n*) of gels (biological replicates, 25-30 animals each), show changes in protein expression in the *D. pulex* clone G after the acute exposure of control animals (20 °C, ad libitum feeding) (blue spots; *n* = 5) to (a) 24 h (orange spots; *n* = 4) or (c) 48 h (orange spots; *n* = 4) of starvation (*T* = 20 °C). Red or green spot IDs mark significantly up- or downregulated proteins (t-tests, *P* < 0.05; see Table 4). The scatter plots show changes in expression level (V_relative_, relative spot volume) between control and starving animals (b, 24 h; d, 48 h) of significantly (large circles and letters) or non-significantly (small circles) up- or down-regulated proteins (data from a or c). Proteins, which were upregulated under starvation stress, are found above the diagonal line. (PDF 126 kb)

Additional file 4: **Figure S3.** Two-dimensional protein gels from the *D. pulex* clone M under starvation stress. The 2D gels, which are fusion (averaged) images from a varying number (*n*) of gels (biological replicates, 25-30 animals each), show changes in protein expression in the *D. pulex* clone M after the acute exposure of control animals (20 °C, ad libitum feeding) (blue spots; *n* = 4) to (a) 24 h (orange spots; *n* = 4) or (c) 48 h (orange spots; *n* = 5) of starvation (T = 20 °C). Red or green spot IDs mark significantly up- or downregulated proteins (t-tests, *P* < 0.05; see Table 4). See Additional file 3: Figure S2 for further explanations. (PDF 127 kb)

Additional file 5: **Figure S4.** Two-dimensional protein gel from the *D. pulex* clone G under heat-and-starvation stress. The 2D gel, which is a fusion (averaged) image from a varying number (*n*) of gels (biological replicates, 25-30 animals each), shows changes in protein expression in the *D. pulex* clone G after the acute exposure of control animals (20 °C, ad libitum feeding) (blue spots; *n* = 5) to (a) 24 h (orange spots; *n* = 5) of heat-and-starvation stress (*T* = 30 °C, starvation). (Clone G did not survive 48 h of heat-and-starvation stress.) Red or green spot IDs mark significantly up- or downregulated proteins (t-tests, *P* < 0.05; see Table 5). The scatter plot shows changes in expression level (V_relative_, relative spot volume) between control animals and animals exposed to heat-and-starvation stress (b, 24 h) of significantly (large circles and letters) or non-significantly (small circles) up- or down-regulated proteins (data from a). Proteins, which were upregulated under heat-and-starvation stress, are found above the diagonal line. (PDF 152 kb)

Additional file 6: **Figure S5.** Two-dimensional protein gels from the *D. pulex* clone M under heat-and-starvation stress. The 2D gels, which are fusion (averaged) images from a varying number (*n*) of gels (biological replicates, 25-30 animals each), show changes in protein expression in the *D. pulex* clone M after the acute exposure of control animals (20 °C, ad libitum feeding) (blue spots; *n* = 4) to (a) 24 h (orange spots; *n* = 5) or (c) 48 h (orange spots; *n* = 7) of heat-and-starvation stress (T = 30 °C, starvation). Red or green spot IDs mark significantly up- or downregulated proteins (t-tests, *P* < 0.05; see Table 5). The scatter plots show changes in expression level (V_relative_, relative spot volume) between control animals and animals exposed to heat-and-starvation stress (b, 24 h; d, 48 h) of significantly (large circles and letters) or non-significantly (small circles) up- or down-regulated proteins (data from a or c). Proteins, which were upregulated under heat-and-starvation stress, are found above the diagonal line. (PDF 194 kb)

Additional file 7: **Figure S6.** Experimental design. (PDF 144 kb)

Additional file 8: **Table S2.** Labeling design of the microarrays. (PDF 83 kb)

## Abbreviations

CHR: Cellular homeostasis response
CSR: Cellular stress response
DEG(s): Differentially expressed gene(s)
DEP(s): Differentially expressed protein(s)
FDR: False discovery rate
FKBP: FK506 binding protein
FOG: Fuzzy orthologous groups
HSP: Heat shock protein
JGI: JOINT Genome Institute
KOG: Eukaryotic orthologous groups
KOGs: KOG-identified DEGs
LRR: Leucine-rich repeat
MDS: Multidimensional scaling
OCLTT: Oxygen-and-capacity-limited temperature tolerance
ROS: Reactive oxygen species
SOD: Superoxide dismutase
UDP: Uridine diphosphate
UPR: Unfolded protein response
VTG: Vitellogenin
VTG-SOD: Vitellogenin fused to superoxide dismutase
β-TrCP: β-transducin repeat-containing protein

## References

[1] Sell A (1998). Adaptation to oxygen deficiency: contrasting patterns of haemoglobin synthesis in two coexisting *Daphnia* species. Comp Biochem Physiol A.

[2] Weider LJ (1985). Spatial and temporal genetic heterogeneity in a natural *Daphnia* population. J Plankton Res.

[3] Lampert W, Fleckner W, Rai H, Taylor BE (1986). Phytoplankton control by grazing zooplankton: a study on the spring clear-water phase. Limnol Oceanogr.

[4] Hochachka PW, Somero GN (2002). Biochemical adaptation.

[5] Becker D, Brinkmann BF, Zeis B, Paul RJ (2011). Acute changes in temperature or oxygen availability induce ROS fluctuations in *Daphnia magna* linked with fluctuations of reduced and oxidized glutathione, catalase activity and gene (haemoglobin) expression. Biol Cell.

[6] Pörtner HO, Knust R (2007). Climate change affects marine fishes through the oxygen limitation of thermal tolerance. Science.

[7] Kültz D (2005). Molecular and evolutionary basis of the cellular stress response. Annu Rev Physiol.

[8] Hebert PDN, Peters RH, De Bernardi R (1987). Genetics of *Daphnia*. *Daphnia*. Mem Inst Ital Idrobiol.

[9] Weider LJ, Makino W, Acharya K, Glenn KL, Kyle M, Urabe J (2005). Genotype x environment interactions, stoichiometric food quality effects, and clonal coexistence in *Daphnia pulex*. Oecologia.

[10] Paul RJ, Mertenskötter A, Pinkhaus O, Pirow R, Gigengack U, Buchen I (2012). Seasonal and interannual changes in water temperature affect the genetic structure of a *Daphnia* assemblage (*D. longispina* complex) through genotype-specific thermal tolerances. Limnol Oceanogr.

[11] Lockwood BL, Sanders JG, Somero GN (2010). Transcriptomic responses to heat stress in invasive and native blue mussels (genus *Mytilus*): molecular correlates of invasive success. J Exp Biol.

[12] Zhang G, Fang X, Guo X, Li L, Luo R, Xu F (2012). The oyster genome reveals stress adaptation and complexity of shell formation. Nature.

[13] Sorensen JG, Nielsen MM, Kruhoffer M, Justesen J, Loeschcke V (2005). Full genome gene expression analysis of the heat stress response in *Drosophila melanogaster*. Cell Stress Chaperon.

[14] Spriggs KA, Bushell M, Willis AE (2010). Translational regulation of gene expression during conditions of cell stress. Mol Cell.

[15] Shalgi R, Hurt JA, Krykbaeva I, Taipale M, Lindquist S, Burge CB (2013). Widespread regulation of translation by elongation pausing in heat shock. Mol Cell.

[16] Lackner DH, Schmidt MW, Wu S, Wolf DA, Bähler J (2012). Regulation of transcriptome, translation, and proteome in response to environmental stress in fission yeast. Genome Biol.

[17] Lee MV, Topper SE, Hubler SL, Hose J, Wenger CD, Coon JJ (2011). A dynamic model of proteome changes reveals new roles for transcript alteration in yeast. Mol Syst Biol.

[18] Klumpen E, Hoffschröer N, Zeis B, Gigengack U, Dohmen E, Paul RJ (2017). Reactive oxygen species (ROS) and the heat stress response of *Daphnia pulex*: ROS-mediated activation of hypoxia-inducible factor 1 (HIF-1) and heat shock factor 1 (HSF-1) and the clustered expression of stress genes. Biol Cell.

[19] Paul RJ, Zeis B, Lamkemeyer T, Seidl M, Pirow R (2004). Control of oxygen transport in the microcrustacean *Daphnia*: regulation of haemoglobin expression as central mechanism of adaptation to different oxygen and temperature conditions. Acta Physiol Scand.

[20] Colbourne JK, Pfrender ME, Gilbert D, Thomas WK, Tucker A, Oakley TH (2011). The ecoresponsive genome of *Daphnia pulex*. Science.

[21] Gerke P, Börding C, Zeis B, Paul RJ (2011). Adaptive haemoglobin gene control in *Daphnia pulex* at different oxygen and temperature conditions. Comp Biochem Physiol A Mol Integr Physiol.

[22] Kang CB, Hong Y, Dhe-Paganon S, Yoon HS (2008). FKBP family proteins: immunophilins with versatile biological functions. Neurosignals.

[23] Tatusov RL, Fedorova ND, Jackson JD, Jacobs AR, Kiryutin B, Koonin EV, Krylov DM, Mazumder R, Mekhedov SL, Nikolskaya AN, Rao BS, Smirnov S, Sverdlov AV, Vasudevan S, Wolf YI, Yin JJ, Natale DA (2003). The COG database: an updated version includes eukaryotes. BMC Bioinformatics.

[24] Kobe B, Kajava AV (2001). The leucine-rich repeat as a protein recognition motif. Curr Opin Struct Biol.

[25] Dölling R, Becker D, Hawat S, Koch M, Schwarzenberger A, Zeis B (2016). Adjustments of serine proteases of *Daphnia pulex* in response to temperature changes. Comp Biochem Physiol B Biochem Mol Biol.

[26] Busino L, Donzelli M, Chiesa M, Guardavaccaro D, Ganoth D, Dorrello NV (2003). Degradation of Cdc25A by β-TrCP during S phase and in response to DNA damage. Nature.

[27] Dorrello NV, Peschiaroli A, Guardavaccaro D, Colburn NH, Sherman NE, Pagano M (2006). S6K1- and β-TRCP-mediated degradation of PDCD4 promotes protein translation and cell growth. Science.

[28] Chen CH (2012). Activation and detoxification enzymes: functions and implications.

[29] Silverstein RL, Li W, Park YM, Rahaman SO (2010). Mechanisms of cell signaling by the scavenger receptor CD36: implications in atherosclerosis and thrombosis. Trans Am Clin Climatol Assoc.

[30] Gess B, Hofbauer KH, Wenger RH, Lohaus C, Meyer HE, Kurtz A (2003). The cellular oxygen tension regulates expression of the endoplasmic oxidoreductase ERO1-Lα. FEBS J.

[31] Kezhong Zhang K, Randal J, Kaufman RJ (2006). The unfolded protein response. Neurology.

[32] Martin J, Horwich AL, Hartl FU (1992). Prevention of protein denaturation under heat stress by the chaperonin Hsp60. Science.

[33] Vargas-Parada L, Solis CF, Laclette JP (2001). Heat shock and stress response of *Taenia solium* and *T. crassiceps* (Cestoda). Parasitology.

[34] Koll H, Guiard B, Rassow J, Ostermann J, Horwich AL, Neupert W (1992). Antifolding activity of hsp60 couples protein import into the mitochondrial matrix with export to the intermembrane space. Cell.

[35] Kaufman BA, Kolesar JE, Perlman PS, Butow RA (2003). A function for the mitochondrial chaperonin Hsp60 in the structure and transmission of mitochondrial DNA nucleoids in *Saccharomyces cerevisiae*. J Cell Biol.

[36] Itoh H, Komatsuda A, Ohtani H, Wakui H, Imai H, Sawada K (2002). Mammalian HSP60 is quickly sorted into the mitochondria under conditions of dehydration. Eur J Biochem.

[37] Buttgereit F, Brand MD (1995). A hierarchy of ATP-consuming processes in mammalian cells. Biochem J.

[38] Kerr MS (1969). The hemolymph proteins of the blue crab, *Callinectes sapidus*: II. A lipoprotein serologically identical to oocyte lipovitellin. Dev Biol.

[39] Storey KB (1996). Oxidative stress: animal adaptations in nature. Braz J Med Biol Res.

[40] Kato Y, Tokishita S, Ohta T, Yamagata HA (2004). Vitellogenin chain containing a superoxide dismutase-like domain is the major component of yolk proteins in cladoceran crustacean *Daphnia magna*. Gene.

[41] Chen S, Chen DF, Yang F, Nagasawa H, Yang WJ (2011). Characterization and processing of superoxide dismutase-fused vitellogenin in the diapause embryo formation: a special developmental pathway in the brine shrimp, *Artemia parthenogenetica*. Biol Reprod.

[42] Ellington WR (2001). Evolution and physiological roles of phosphagen systems. Annu Rev Physiol.

[43] Ballatori N, Krance SM, Marchan R, Hammond CL (2009). Plasma membrane glutathione transporters and their roles in cell physiology and pathophysiology. Mol Asp Med.

[44] Beyenbach KW, Wieczorek H (2006). The V-type H+ ATPase: molecular structure and function, physiological roles and regulation. J Exp Biol.

[45] Gelebart P, Opas M, Michalak M (2005). Calreticulin, a Ca^2+^-binding chaperone of the endoplasmic reticulum. Int J Biochem Cell B.

[46] Conway EM, Liu L, Nowakowski B, Steiner-Mosonyi M, Ribeiro SP, Michalak M (1995). Heat shock-sensitive expression of calreticulin. In vitro and in vivo up-regulation. J Biol Chem.

[47] Jethmalani SM, Henle KJ (1998). Calreticulin associates with stress proteins: implications for chaperone function during heat stress. J Cell Biochem.

[48] Prasad SC, Soldatenkov VA, Kuettel MR, Thraves PJ, Zou X, Dritschilo A (1999). Protein changes associated with ionizing radiation-induced apoptosis in human prostate epithelial tumor cells. Electrophoresis.

[49] Tarr JM, Young PJ, Morse R, Shaw DJ, Haigh R, Petrov PG (2010). A mechanism of release of calreticulin from cells during apoptosis. J Mol Biol.

[50] Hatahet F, Ruddock LW (2007). Substrate recognition by the protein disulfide isomerases. FEBS J.

[51] Hebert PDN, Beaton MJ (1989). Methodologies for allozyme analysis using cellulose acetate electrophoresis. A practical handbook.

[52] Matthes M (2004). Low genotypic diversity in a *Daphnia pulex* population in a biomanipulated lake: the lack of vertical and seasonal variability. Hydrobiologia.

[53] Elendt BP, Bias WR (1990). Trace nutrient deficiency in *Daphnia magna* cultured in standard medium for toxicity testing. Effects of the optimization of culture conditions on life history parameters of D. Magna. Water Res.

[54] Lopez JA, Colbourne JK (2011). Dual-labeled expression microarray protocol for high-throughput genomic investigations. CGB technical report 2011-02.

[55] Ihaka R, Gentleman R (1996). R a language for data analysis and graphics. J Comput Graph Stat.

[56] Gentleman RC, Carey VJ, Bates DM, Bolstad B, Dettling M, Ellis B (2004). Bioconductor: open software development for computational biology and bioinformatics. Genome Biol.

[57] Smyth GK (2004). Linear models and empirical bayes methods for assessing differential expression in microarray experiments. Stat Appl Genet Mol Biol.

[58] Kendziorski CM, Newton MA, Lan H, Gould MN (2003). On parametric empirical Bayes methods for comparing multiple groups using replicated gene expression profiles. Statist Med.

[59] *Daphnia pulex* genome assembly v1.1. http://wfleabase.org/genome/Daphnia_pulex/. Accessed 7 May 2018.

[60] Benjamini Y, Hochberg Y (1995). Controlling the false discovery rate: a practical and powerful approach to multiple testing. J Roy Statist Soc Ser B.

[61] Koonin EV, Fedorova ND, Jackson JD, Jacobs AR, Krylov DM, Makarova KS (2004). A comprehensive evolutionary classification of proteins encoded in complete eukaryotic genomes. Genome Biol.

[62] *Daphnia pulex* (JGI Genome Portal: KOG classification). https://genome.jgi.doe.gov/cgi-bin/kogBrowser?db=Dappu1. Accessed 7 May 2018.

[63] The R Project for Statistical Computing. https://www.r-project.org/. Accessed 7 May 2018.

[64] Bradford MMA (1976). Rapid and sensitive method for the quantitation of microgram quantities of protein utilizing the principle of protein-dye binding. Anal Biochem.

[65] Schwerin S, Zeis B, Lamkemeyer T, Paul RJ, Koch M, Madlung J (2009). Acclimatory responses of the *Daphnia pulex* proteome to environmental changes. II. Chronic exposure to different temperatures (10 and 20 degrees C) mainly affects protein metabolism. BMC Physiol.

[66] Lamanda A, Zahn A, Roder D, Langen H (2004). Improved ruthenium II tris(bathophenantroline disulfonate) staining and destaining protocol for a better signal-to-background ratio and improved baseline resolution. Proteomics.

[67] Shevchenko A, Tomas H, Havlis J, Olsen JV, Mann M (2007). In-gel digestion for mass spectrometric characterization of proteins and proteomes. Nat Protocols.

[68] JGI genome portal: *Daphnia pulex*. https://genome.jgi.doe.gov/portal/Dappu1/Dappu1.download.ftp.html. Accessed 7 May 2018.

[69] Geer LY, Markey SP, Kowalak JA, Wagner L, Xu M, Maynard DM (2004). Open mass spectrometry search algorithm. J Proteome Res.

[70] Craig R, Beavis RC (2004). TANDEM: matching proteins with tandem mass spectra. Bioinformatics.

[71] X! TANDEM Spectrum Modeler. https://www.thegpm.org/TANDEM/index.html. Accessed 7 May 2018.

[72] Specht M, Kuhlgert S, Fufezan C, Hippler M (2011). Proteomics to go: Proteomatic enables the user-friendly creation of versatile MS/MS data evaluation workflows. Bioinformatics.

[73] Petersen TN, Brunak S, von Heijne G, Nielsen H (2011). SignalP 4.0: discriminating signal peptides from transmembrane regions. Nat Methods.

[74] Gasteiger E, Hoogland C, Gattiker A, Duvaud S, Wilkins MR, Appel RD, et al. Protein identification and analysis tools on the ExPASy server. In: Walker JM, editor. The proteomics protocols handbook. Totowa: Humana Press Inc; 2005. p. 571–607.

[75] Romero-Calvo I, Ocon B, Martinez-Moya P, Suarez MD, Zarzuelo A, Martinez-Augustin O (2010). Reversible Ponceau staining as a loading control alternative to actin in western blots. Anal Biochem.

[76] Mikulski A, Grzesiuk M, Kloc M, Pijanowska J (2009). Heat shock proteins in *Daphnia* detected using commercial antibodies: description and responsiveness to thermal stress. Chemoecology.

[77] Home - *Daphnia* Genomics Consortium - Collaboration Wiki. https://wiki.cgb.indiana.edu/display/DGC/Home. Accessed 7 May 2018.

