# Supplementary material for: The transcriptomic and proteomic responses of Daphnia pulex to changes in temperature and food supply comprise environment-specific and clone-specific elements
Source: BMC Genomics. 2018 May 21;19:376. doi: 10.1186/s12864-018-4742-6 (PMC5963186; doi:10.1186/s12864-018-4742-6)
Supplement: Supplementary file 1 — Table S1. KOG-identified DEGs shared by the contrasts under acute heat stress. (PDF 125 kb) [file 12864_2018_4742_MOESM1_ESM.pdf]

Table S1. KOG-identified DEGs shared by the contrasts under acute heat stress.

N	Gene	L2h	L4h	L8h	K ID	Description	Cat
1	18244	-1.06	-1.91	-1.17	0107	Alternative_splicing_factor_SRp20/9G8_(RRM_superfamily)	[A]
2	128657	1.38	1.45	1.53	1744	Histone_H2B	[B]
3	320454	1.00	0.99	1.30	1744	Histone_H2B	[B]
4	98275	2.24	1.70	0.98	2084	Predicted_histone_tail_methylase_containing_SET_domain	[B]
5	239494	2.04	1.89	1.32	2084	Predicted_histone_tail_methylase_containing_SET_domain	[B]
6	194440	1.41	1.54	1.54	2084	Predicted_histone_tail_methylase_containing_SET_domain	[B]
7	222354	1.35	1.32	1.48	0537	Cytochrome_b5	[C]
8	309167	1.72	2.04	1.54	3573	Cysteine_endopeptidases._,Caspase._apoptotic_cysteine_protease	[D]
9	310275	-0.93	-0.99	-1.05	1304	Amino_acid_transporters	[E]
10	237405	-0.94	-1.09	-0.86	1287	Amino_acid_transporters	[E]
11	237404	-1.36	-1.32	-1.28	1287	Amino_acid_transporters	[E]
12	241460	1.11	2.11	1.21	3627	Chymotrypsin._,Trypsin	[E]
13	304512	-2.13	-2.25	-2.23	3627	Chymotrypsin._,Trypsin	[E]
14	319507	-2.29	-2.11	-1.84	3627	Chymotrypsin._,Trypsin	[E]
15	46936	-1.26	-1.16	-1.29	1237	H+/oligopeptide_symporter	[E]
16	26258	-2.53	-2.55	-0.88	3627	Serine_endopeptidases._,Trypsin	[E]
17	317407	-0.94	-1.10	-0.95	1383	Sphinganine-1-phosphate_aldolase._Sphingoglycolipid_metabolism,Glutamate_decarboxylase/sphingosine_phosphate_lyase	[E]
18	49241	-1.19	-2.02	-1.64	3627	Trypsin	[E]
19	307264	-1.20	-1.21	-0.85	3627	Trypsin	[E]
20	248152	-1.31	-1.54	-1.60	3627	Trypsin	[E]
21	52244	-2.20	-2.67	-1.16	3627	Trypsin	[E]
22	231482	-1.26	-1.12	-1.53	3627	Trypsin._,Trypsin	[E]
23	305925	-1.75	-1.74	-1.93	3627	Trypsin._,Trypsin	[E]
24	302859	-1.62	-1.81	-1.57	1046	Membrane_alanine_aminopeptidase._Glutathione_metabolism,Puromycin-sensitive_aminopeptidase_and_related_aminopeptidases	[EO]
25	196131	-1.47	-2.23	-1.00	1046	Puromycin-sensitive_aminopeptidase_and_related_aminopeptidases	[EO]
26	116436	-1.61	-1.03	-1.61	4238	Bifunctional_ATP_sulfurylase/adenosine_5'-phosphosulfate_kinase	[F]
27	64687	-1.23	-0.95	-1.12	2212	Alpha-amylase	[G]
28	326098	-1.34	-1.03	-1.40	0496	Beta-galactosidase._Glycosaminoglycan_degradation,Beta-galactosidase._Galactose_metabolism,Beta-galactosidase._N-Glycan_degradation,Beta-galactosidase._Glycerolipid_metabolism,Beta-galactosidase._Sphingoglycolipid_metabolism,Beta-galactosidase	[G]
29	318905	-1.65	-1.97	-2.08	0626	Beta-glucosidase._Starch_and_sucrose_metabolism,Beta-glucosidase._Cyanoamino_acid_metabolism,Beta-glucosidase._Flavonoids._stilbene_and_lignin_biosynthesis,Beta-glucosidase._lactase_phlorizinhydrolase._and_related_proteins	[G]
30	311672	-1.03	-0.86	-1.04	2230	Beta-mannosidase._N-Glycan_degradation,Predicted_beta-mannosidase	[G]
31	310994	-1.01	-0.77	-1.20	2806	Chitinase	[G]
32	312136	-1.08	-1.11	-0.96	1065	Maltase_glucoamylase_and_related_hydrolases._glycosyl_hydrolase_family_31	[G]
33	64229	-1.70	-1.88	-1.67	1065	Maltase_glucoamylase_and_related_hydrolases._glycosyl_hydrolase_family_31	[G]
34	301986	-1.45	-1.41	-0.98	2532	Permease_of_the_major_facilitator_superfamily	[G]
35	58734	-1.52	-1.52	-1.24	1330	Sugar_transporter/spinster_transmembrane_protein	[G]
36	325973	-2.55	-2.56	-1.26	1330	Sugar_transporter/spinster_transmembrane_protein	[G]
37	330188	-2.47	-0.84	-2.14	1192	UDP-glucuronosyl_and_UDP-glucosyl_transferase	[GC]
38	317312	-0.94	-1.26	-0.92	1192	UDP-glucuronosyltransferase._Porphyrin_and_chlorophyll_metabolism,UDP-glucuronosyltransferase._Androgen_and_estrogen_metabolism,UDP-glucuronosyltransferase._Starch_and_sucrose_metabolism,UDP-glucuronosyltransferase._Pentose_and_glucuronate_interconversio	[GC]
39	299672	-0.95	-1.04	-1.09	4254	Phytoene_desaturase	[H]
40	200149	-1.50	-1.44	-1.60	1014	17_beta-hydroxysteroid_dehydrogenase_type_3._HSD17B3	[I]
41	316454	1.32	1.18	1.30	1176	4-coumarate--CoA_ligase._Flavonoids._stilbene_and_lignin_biosynthesis,Acyl-CoA_synthetase	[I]
42	307236	-0.88	-1.12	-1.04	1471	Phosphatidylinositol_transfer_protein_SEC14_and_related_proteins	[I]
43	300303	-1.41	-1.44	-1.02	1471	Phosphatidylinositol_transfer_protein_SEC14_and_related_proteins	[I]
44	314180	-1.35	-1.13	-1.20	1471	Protein-tyrosine-phosphatase._,Phosphatidylinositol_transfer_protein_SEC14_and_related_proteins	[I]
45	306834	1.59	1.45	1.43	3119	Basic_region_leucine_zipper_transcription_factor	[K]
46	300656	0.84	0.95	0.85	1561	CCAAT-binding_factor._subunit_B_(HAP2)	[K]
47	329036	0.95	1.72	1.72	4282	Transcription_factor_GT-2_and_related_proteins._contains_trihelix_DNA-binding/SANT_domain	[K]
48	259955	1.92	1.55	1.82	4029	Transcription_factor_HAND2/Transcription_factor_TAL1/TAL2/LYL1	[K]
49	8458	2.51	2.36	1.74	3898	Transcription_factor_NeuroD_and_related_HTH_proteins	[K]
50	209021	-1.75	-1.31	-1.56	2248	Acting_on_ester_bonds._,3'-5'_exonuclease	[L]
51	249011	1.07	0.88	0.70	4177	Ankyrin	[M]
52	234666	1.91	1.91	1.64	4824	Apolipoprotein_D/Lipocalin	[M]
53	230313	1.65	1.68	0.92	2160	Armadillo/beta-catenin-like_repeat-containing_protein	[O]
54	93694	-1.78	-2.14	-1.61	3714	Astacin._,Meprin_A_metalloprotease	[O]
55	189045	-0.88	-0.75	-1.17	1339	Cathepsin_D._,Aspartyl_protease	[O]
56	227643	-1.64	-2.06	-2.06	1339	Cathepsin_D._,Aspartyl_protease	[O]
57	301074	1.46	1.11	0.84	0356	Chaperonin_ATPase._,Mitochondrial_chaperonin._Cpn60/Hsp60p	[O]

58	309186	1.87	1.61	1.02	0020	Endoplasmic_reticulum_glucose-regulated_protein_(GRP94/endoplasmin)._HSP90_family	[O]
59	303608	1.83	1.30	1.04	0543	FKBP-type_peptidyl-prolyl_cis-trans_isomerase	[O]
60	204206	1.64	0.94	0.89	0543	FKBP-type_peptidyl-prolyl_cis-trans_isomerase	[O]
61	316498	1.38	0.78	0.70	1695	Glutathione_transferase._Glutathione_metabolism,Glutathione_S-transferase	[O]
62	210571	1.36	1.11	1.18	1695	Glutathione_transferase._Glutathione_metabolism,Glutathione_S-transferase	[O]
63	240930	1.75	1.56	1.15	0714	In_phosphorous-containing_anhydrides._Starch_and_sucrose_metabolism,In_phosphorous-containing_anhydrides._Folate_biosynthesis,Molecular_chaperone_(DnaJ_superfamily)	[O]
64	332162	-2.07	-2.47	-2.27	3714	Meprin_A_metalloprotease	[O]
65	304197	2.01	1.53	1.24	0714	Molecular_chaperone_(DnaJ_superfamily)	[O]
66	305330	1.82	1.54	1.23	0712	Molecular_chaperone_(DnaJ_superfamily)	[O]
67	265141	2.21	1.47	1.26	0019	Molecular_chaperone_(HSP90_family)	[O]
68	99491	2.16	1.27	1.35	0019	Molecular_chaperone_(HSP90_family)	[O]
69	99736	2.16	1.23	1.42	0019	Molecular_chaperone_(HSP90_family)	[O]
70	66705	2.15	1.16	1.48	0019	Molecular_chaperone_(HSP90_family)	[O]
71	116816	2.13	1.21	1.35	0019	Molecular_chaperone_(HSP90_family)	[O]
72	302452	2.05	1.27	1.10	0019	Molecular_chaperone_(HSP90_family)	[O]
73	113325	2.05	1.29	1.14	0019	Molecular_chaperone_(HSP90_family)	[O]
74	66707	2.02	1.03	1.53	0019	Molecular_chaperone_(HSP90_family)	[O]
75	273773	1.97	1.37	1.18	0019	Molecular_chaperone_(HSP90_family)	[O]
76	116313	1.94	1.28	1.30	0019	Molecular_chaperone_(HSP90_family)	[O]
77	119333	1.93	1.21	1.39	0019	Molecular_chaperone_(HSP90_family)	[O]
78	65101	1.91	1.06	1.35	0019	Molecular_chaperone_(HSP90_family)	[O]
79	70250	1.90	0.97	1.38	0019	Molecular_chaperone_(HSP90_family)	[O]
80	118578	1.79	1.15	1.02	0019	Molecular_chaperone_(HSP90_family)	[O]
81	8016	1.75	1.22	1.12	0019	Molecular_chaperone_(HSP90_family)	[O]
82	28667	1.55	1.11	0.90	0019	Molecular_chaperone_(HSP90_family)	[O]
83	307965	1.39	1.06	0.72	0019	Molecular_chaperone_(HSP90_family)	[O]
84	229110	2.13	1.44	0.98	0103	Molecular_chaperones_HSP105/HSP110/SSE1._HSP70_superfamily	[O]
85	34365	1.72	0.81	0.71	0103	Molecular_chaperones_HSP105/HSP110/SSE1._HSP70_superfamily	[O]
86	302856	2.98	2.13	1.99	0101	Molecular_chaperones_HSP70/HSC70._HSP70_superfamily	[O]
87	227800	1.78	1.75	1.36	0101	Molecular_chaperones_HSP70/HSC70._HSP70_superfamily	[O]
88	304017	-0.87	-0.93	-1.12	0549	Peptidylprolyl_isomerase._,FKBP-type_peptidyl-prolyl_cis-trans_isomerase	[O]
89	307787	-0.90	-1.45	-0.78	1695	Prostaglandin-D_synthase._Prostaglandin_and_leukotriene_metabolism,Glutathione_S-transferase	[O]
90	218095	2.04	1.07	1.06	0191	Protein_disulfide_isomerase._,Thioredoxin/protein_disulfide_isomerase	[O]
91	303754	1.91	1.70	1.83	4127	Renal_dipeptidase	[O]
92	302623	1.93	1.82	1.14	1866	Ubiquitin_carboxyl-terminal_hydrolase	[O]
93	302917	2.39	1.93	1.47	4151	Myosin_assembly_protein/sexual_cycle_protein_and_related_proteins	[ODR]
94	257458	1.95	1.62	1.11	4151	Myosin_assembly_protein/sexual_cycle_protein_and_related_proteins	[ODR]
95	307492	1.60	0.78	0.82	1308	Hsp70-interacting_protein_Hip/Transient_component_of_progesterone_receptor_complexes_and_an_Hsp70-binding_protein	[OT]
96	308173	1.36	1.22	0.95	2608	With_a_disulfide_as_acceptor._,Endoplasmic_reticulum_membrane-associated_oxidoreductin_involved_in_disulfide_bond_formation	[OU]
97	327165	1.29	0.95	1.11	0682	Ammonia_permease	[P]
98	226776	-0.97	-0.85	-0.81	1281	Na+/dicarboxylate._Na+/tricarboxylate_and_phosphate_transporters	[P]
99	100474	1.63	1.14	1.25	1052	Glutamate-gated_kainate-type_ion_channel_receptor_subunit_GluR5_and_related_subunits	[PET]
100	49503	1.00	1.00	1.31	1263	L-ascorbate_oxidase._Ascorbate_and_aldarate_metabolism,Multicopper_oxidases	[Q]
101	312948	-1.48	-1.19	-0.83	0061	Transporter._ABC_superfamily_(Breast_cancer_resistance_protein)	[Q]
102	127566	2.27	1.64	1.15	1577	Aldo/keto_reductase_family_proteins	[R]
103	332780	1.39	1.34	1.40	0508	Ankyrin_repeat_protein	[R]
104	17589	0.92	1.13	1.03	2935	Ataxin_3/Josephin	[R]
105	240264	-2.27	-2.51	-1.54	1516	Carboxylesterase_and_related_proteins	[R]
106	48040	-2.56	-2.27	-0.88	1516	Carboxylesterase_and_related_proteins	[R]
107	301975	-2.67	-1.91	-1.49	1516	Carboxylesterase_and_related_proteins	[R]
108	221219	0.93	1.24	0.80	2408	Peroxidase._Flavonoids._stilbene_and_lignin_biosynthesis,Peroxidase._Phenylalanine_metabolism,Peroxidase._Methane_metabolism,Peroxidase/oxygenase	[R]
109	308194	-1.34	-1.65	-2.38	2408	Peroxidase._Flavonoids._stilbene_and_lignin_biosynthesis,Peroxidase._Phenylalanine_metabolism,Peroxidase._Methane_metabolism,Peroxidase/oxygenase	[R]
110	313823	-1.25	-0.93	-0.99	3700	Predicted_acyltransferase	[R]
111	45680	-1.32	-1.05	-1.14	3700	Predicted_acyltransferase	[R]
112	196694	-1.67	-1.40	-1.49	3700	Predicted_acyltransferase	[R]
113	304067	-2.09	-2.81	-0.87	1520	Predicted_alkaloid_synthase/Surface_mucin_Hemomucin	[R]
114	308242	1.19	1.24	1.07	3782	Predicted_membrane_protein._contains_type_II_SA_sequence	[R]
115	341506	1.27	1.05	0.81	2325	Predicted_transporter/transmembrane_protein	[R]
116	39906	1.18	0.84	1.15	0589	Serine/threonine_protein_kinase	[R]
117	195644	-1.17	-1.47	-1.43	1584	Sulfotransferase	[R]
118	34773	1.21	0.88	0.98	0490	Transcription_factor._contains_HOX_domain	[R]
119	306849	2.85	1.80	1.52	4582	Uncharacterized_conserved_protein._contains_ZZ-type_Zn-finger	[R]
120	195101	1.94	1.36	1.35	1667	Zn2+-binding_protein_Melusin/RAR1._contains_CHORD_domain	[R]
121	304793	3.25	3.01	3.74	0281	Beta-TrCP_(transducin_repeats_containing)/Slimb_proteins	[S]
122	190701	1.28	0.84	0.79	0281	Beta-TrCP_(transducin_repeats_containing)/Slimb_proteins	[S]

123	304918	-1.44	-1.47	-1.04	2650	Carboxypeptidase_A._,Zinc_carboxypeptidase	[S]
124	304390	-1.89	-1.87	-1.08	2650	Carboxypeptidase_A._,Zinc_carboxypeptidase	[S]
125	265595	-2.05	-1.60	-1.26	2650	Carboxypeptidase_A._,Zinc_carboxypeptidase	[S]
126	117945	-1.52	-1.33	-0.84	2650	Carboxypeptidase_B._,Zinc_carboxypeptidase	[S]
127	70299	1.06	0.95	1.06	4308	LRR-containing_protein	[S]
128	306071	1.00	0.86	1.08	4308	LRR-containing_protein	[S]
129	223347	-1.23	-1.17	-1.25	2650	Metallocarboxypeptidases._,Zinc_carboxypeptidase	[S]
130	314031	2.60	2.46	2.73	0271	Notchless-like_WD40_repeat-containing_protein	[S]
131	216884	2.26	1.70	1.44	2936	Uncharacterized_conserved_protein	[S]
132	309336	1.01	0.73	1.10	4067	Uncharacterized_conserved_protein	[S]
133	306762	-1.05	-0.98	-1.10	4065	Uncharacterized_conserved_protein	[S]
134	190503	-1.32	-1.34	-1.02	2650	Zinc_carboxypeptidase	[S]
135	194294	-1.47	-1.13	-1.54	2650	Zinc_carboxypeptidase	[S]
136	224487	-1.65	-1.62	-1.54	2650	Zinc_carboxypeptidase	[S]
137	195011	-2.27	-2.31	-1.18	2650	Zinc_carboxypeptidase	[S]
138	319285	2.97	2.04	1.91	4361	BCL2-associated_athanogene-like_proteins_and_related_BAG_family_chaperone_regulators	[T]
139	309106	-1.10	-1.14	-0.81	2232	Ceramidases	[T]
140	104472	1.24	0.91	0.86	1187	DNA-directed_RNA_polymerase._RNA_polymerase,DNA-directed_RNA_polymerase._Purine_metabolism,DNA-directed_RNA_polymerase._Pyrimidine_metabolism,Serine/threonine_protein_kinase	[T]
141	53633	-1.16	-0.98	-0.71	4244	Failed_axon_connections_(fax)_protein/glutathione_S-transferase-like_protein	[T]
142	325477	1.20	1.09	0.79	0200	Fibroblast/platelet-derived_growth_factor_receptor_and_related_receptor_tyrosine_kinases	[T]
143	113087	2.15	1.37	1.15	3533	Inositol_1.4.5-trisphosphate_receptor	[T]
144	307582	-1.87	-2.31	-0.70	3776	Plasma_membrane_glycoprotein_CD36_and_related_membrane_receptors	[T]
145	122791	-2.20	-2.43	-1.00	3776	Plasma_membrane_glycoprotein_CD36_and_related_membrane_receptors	[T]
146	326408	-2.59	-3.15	-0.85	3776	Plasma_membrane_glycoprotein_CD36_and_related_membrane_receptors	[T]
147	307609	-2.94	-2.31	-2.13	3776	Plasma_membrane_glycoprotein_CD36_and_related_membrane_receptors	[T]
148	224629	-1.52	-1.31	-1.68	4293	Predicted_membrane_protein._contains_DoH_and_Cytochrome_b-561/ferric_reductase_transmembrane_domains	[T]
149	322521	-1.97	-0.88	-1.17	2641	Predicted_seven_transmembrane_receptor_-_rhodopsin_family	[T]
150	318354	2.76	2.97	1.96	0583	Serine/threonine_protein_kinase	[T]
151	303931	1.69	1.69	1.48	1027	Serine/threonine_protein_kinase_and_endoribonuclease_ERN1/IRE1._sensor_of_the_unfolded_protein_response_pathway	[T]
152	58766	1.92	1.68	1.38	3577	Smoothened_and_related_G-protein-coupled_receptors	[T]
153	305446	-0.95	-1.02	-0.79	4297	C-type_lectin	[TV]
154	104167	-1.06	-1.78	-1.10	4297	C-type_lectin	[TV]
155	242162	2.26	1.82	1.35	3671	Actin_regulatory_protein_(Wiskott-Aldrich_syndrome_protein)	[TZ]
156	299609	-1.70	-1.71	-1.20	3699	Cytoskeletal_protein_Adducin	[TZ]
157	40590	3.00	2.25	0.99	3773	Adiponutrin_and_related_vesicular_transport_proteins._predicted_alpha/beta_hydrolase	[U]
158	335548	0.78	0.81	1.03	0946	Myosin_ATPase._Purine_metabolism,ER-Golgi_vesicle-tethering_protein_p115	[U]

The table shows the expression changes of the KOG-identified DEGs shared by the contrasts (30°C vs. 20°C) after 2, 4, and 8 h of acute heat stress. From left to right: Numbering (N), gene ID (JGI_V11_gene ID) (Gene), log_2-fold changes after 2, 4, and 8 h of acute heat stress (L2h, L4h, and L8h), KOG ID (K ID), gene description, KOG category (Cat).
