# Supplementary figures and images for: The transcriptomic and proteomic responses of Daphnia pulex to changes in temperature and food supply comprise environment-specific and clone-specific elements
Source: BMC Genomics. 2018 May 21;19:376. doi: 10.1186/s12864-018-4742-6 (PMC5963186; doi:10.1186/s12864-018-4742-6)

Figure S1.

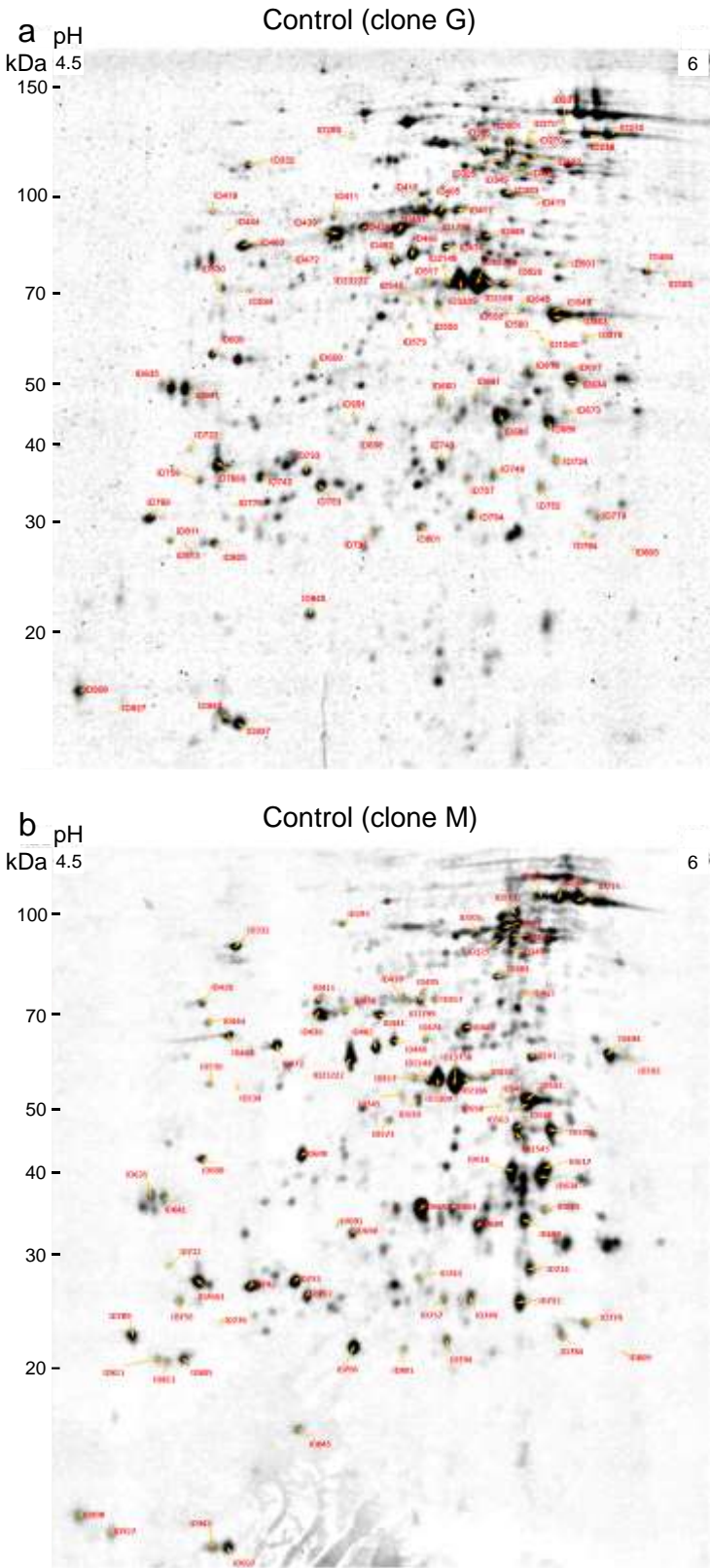

Supplement: Supplementary file 2 — Figure S1. Two-dimensional protein gels from the 20 °C-acclimated D. pulex clones G and M. The RuBPs-stained 2D gels from control animals (20 °C, ad libitum feeding) of (a) the D. pulex clone G [fusion image from n = 5 gels (biological replicates)] and (b) the D. pulex clone M [fusion image from n = 4 gels (biological replicates, 25-30 animals each)] served as reference for the excision of protein spots for mass spectrometry (pI: 4.5-6, molecular mass: 20-150 or 20-100 kDa). Red characters are spot identifiers (IDs). (PDF 118 kb) [file 12864_2018_4742_MOESM2_ESM.pdf]

Figure S2.

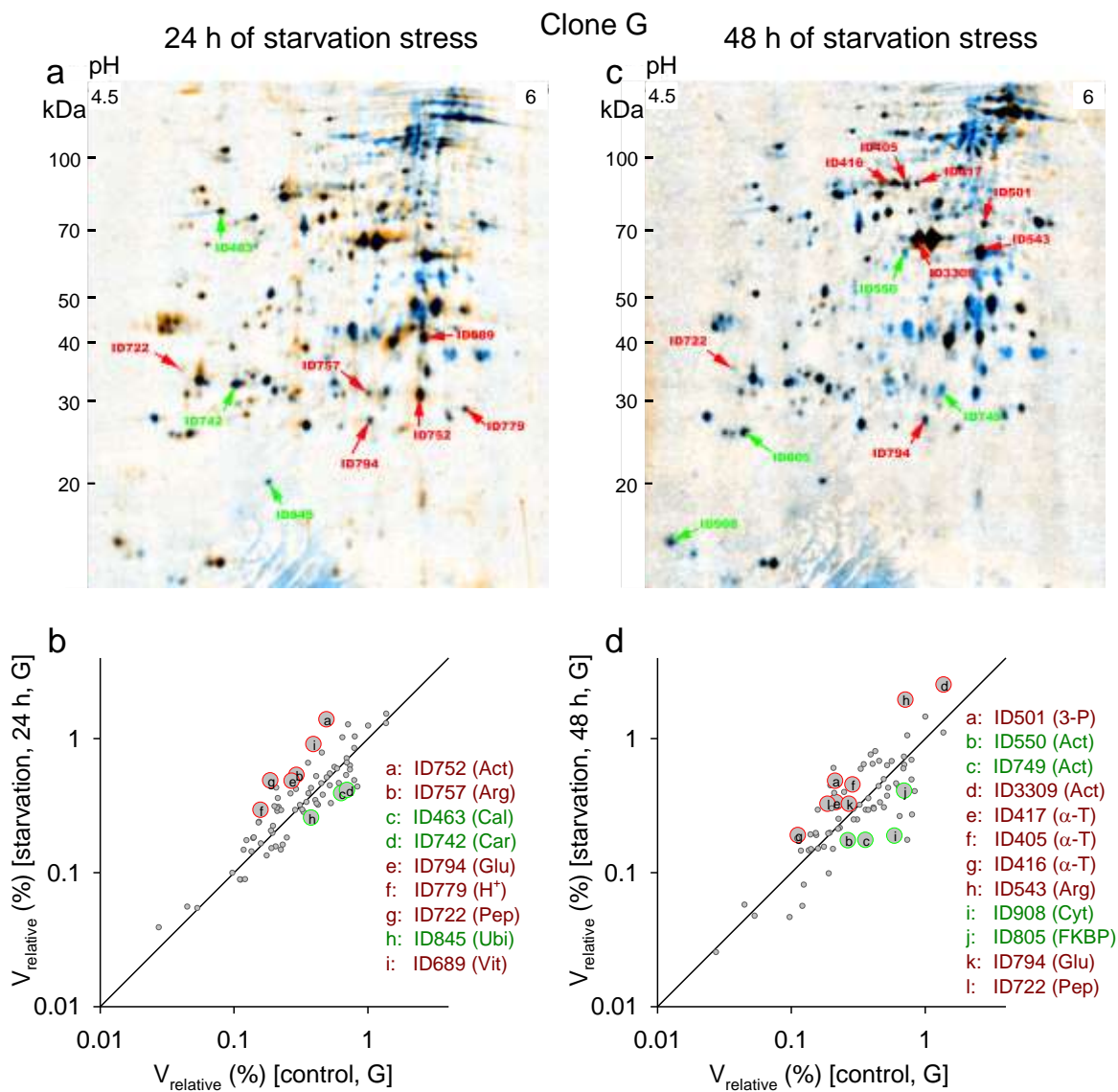

Supplement: Supplementary file 3 — Figure S2. Two-dimensional protein gels from the D. pulex clone G under starvation stress. The 2D gels, which are fusion (averaged) images from a varying number (n) of gels (biological replicates, 25-30 animals each), show changes in protein expression in the D. pulex clone G after the acute exposure of control animals (20 °C, ad libitum feeding) (blue spots; n = 5) to (a) 24 h (orange spots; n = 4) or (c) 48 h (orange spots; n = 4) of starvation (T = 20 °C). Red or green spot IDs mark significantly up- or downregulated proteins (t-tests, P < 0.05; see Table 4). The scatter plots show changes in expression level (Vrelative, relative spot volume) between control and starving animals (b, 24 h; d, 48 h) of significantly (large circles and letters) or non-significantly (small circles) up- or down-regulated proteins (data from a or c). Proteins, which were upregulated under starvation stress, are found above the diagonal line. (PDF 126 kb) [file 12864_2018_4742_MOESM3_ESM.pdf]

Figure S3.

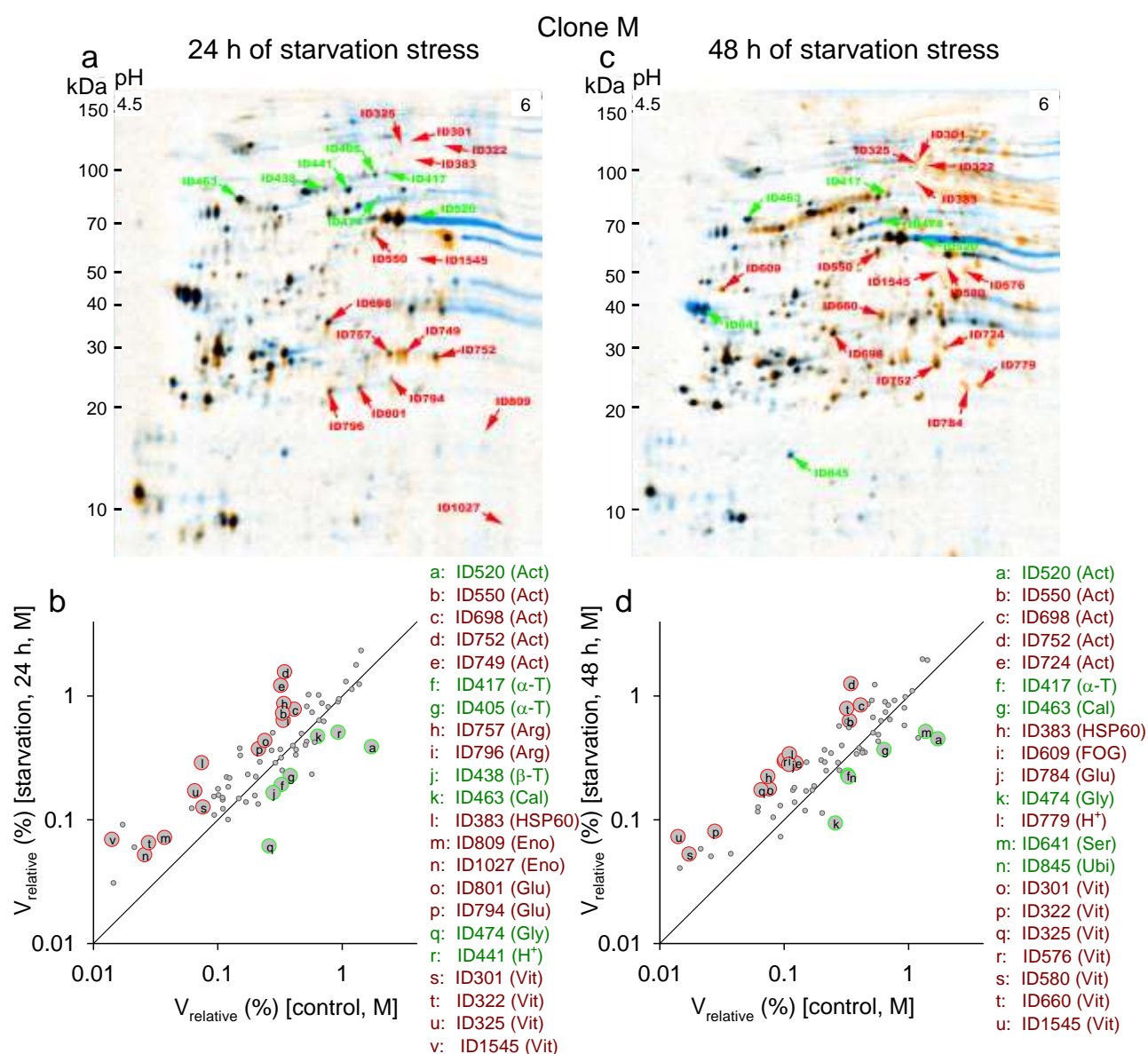

Supplement: Supplementary file 4 — Figure S3. Two-dimensional protein gels from the D. pulex clone M under starvation stress. The 2D gels, which are fusion (averaged) images from a varying number (n) of gels (biological replicates, 25-30 animals each), show changes in protein expression in the D. pulex clone M after the acute exposure of control animals (20 °C, ad libitum feeding) (blue spots; n = 4) to (a) 24 h (orange spots; n = 4) or (c) 48 h (orange spots; n = 5) of starvation (T = 20 °C). Red or green spot IDs mark significantly up- or downregulated proteins (t-tests, P < 0.05; see Table 4). See Additional file 3: Figure S2 for further explanations. (PDF 127 kb) [file 12864_2018_4742_MOESM4_ESM.pdf]

Figure S4.

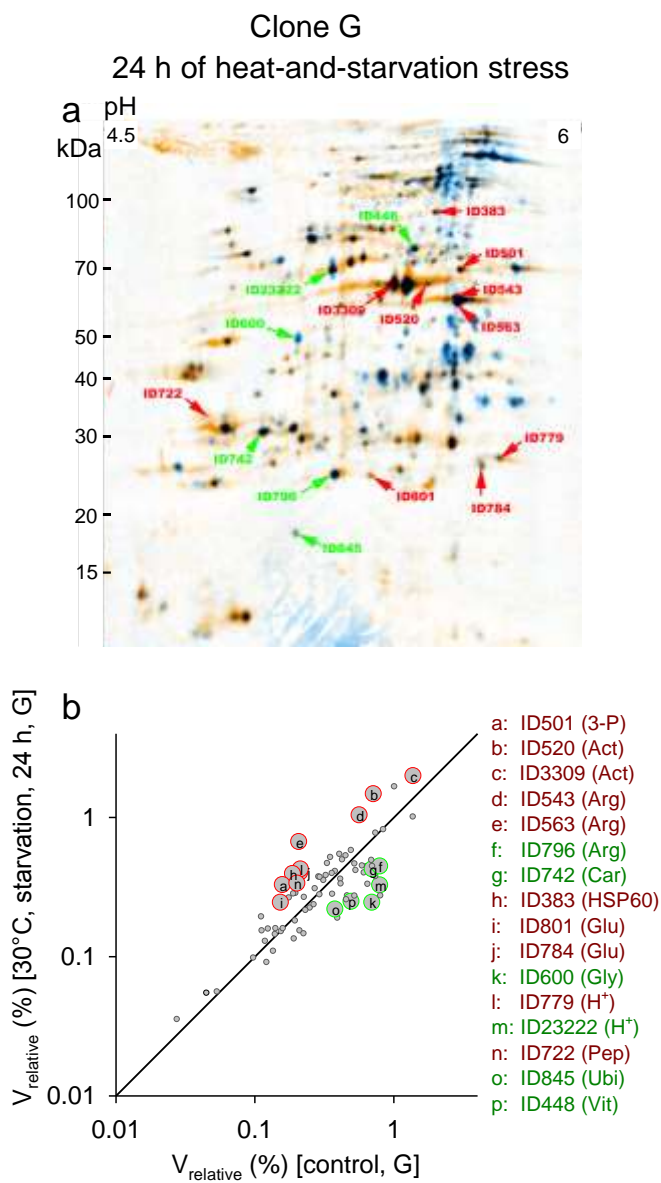

Supplement: Supplementary file 5 — Figure S4. Two-dimensional protein gel from the D. pulex clone G under heat-and-starvation stress. The 2D gel, which is a fusion (averaged) image from a varying number (n) of gels (biological replicates, 25-30 animals each), shows changes in protein expression in the D. pulex clone G after the acute exposure of control animals (20 °C, ad libitum feeding) (blue spots; n = 5) to (a) 24 h (orange spots; n = 5) of heat-and-starvation stress (T = 30 °C, starvation). (Clone G did not survive 48 h of heat-and-starvation stress.) Red or green spot IDs mark significantly up- or downregulated proteins (t-tests, P < 0.05; see Table 5). The scatter plot shows changes in expression level (Vrelative, relative spot volume) between control animals and animals exposed to heat-and-starvation stress (b, 24 h) of significantly (large circles and letters) or non-significantly (small circles) up- or down-regulated proteins (data from a). Proteins, which were upregulated under heat-and-starvation stress, are found above the diagonal line. (PDF 152 kb) [file 12864_2018_4742_MOESM5_ESM.pdf]

## Clone M

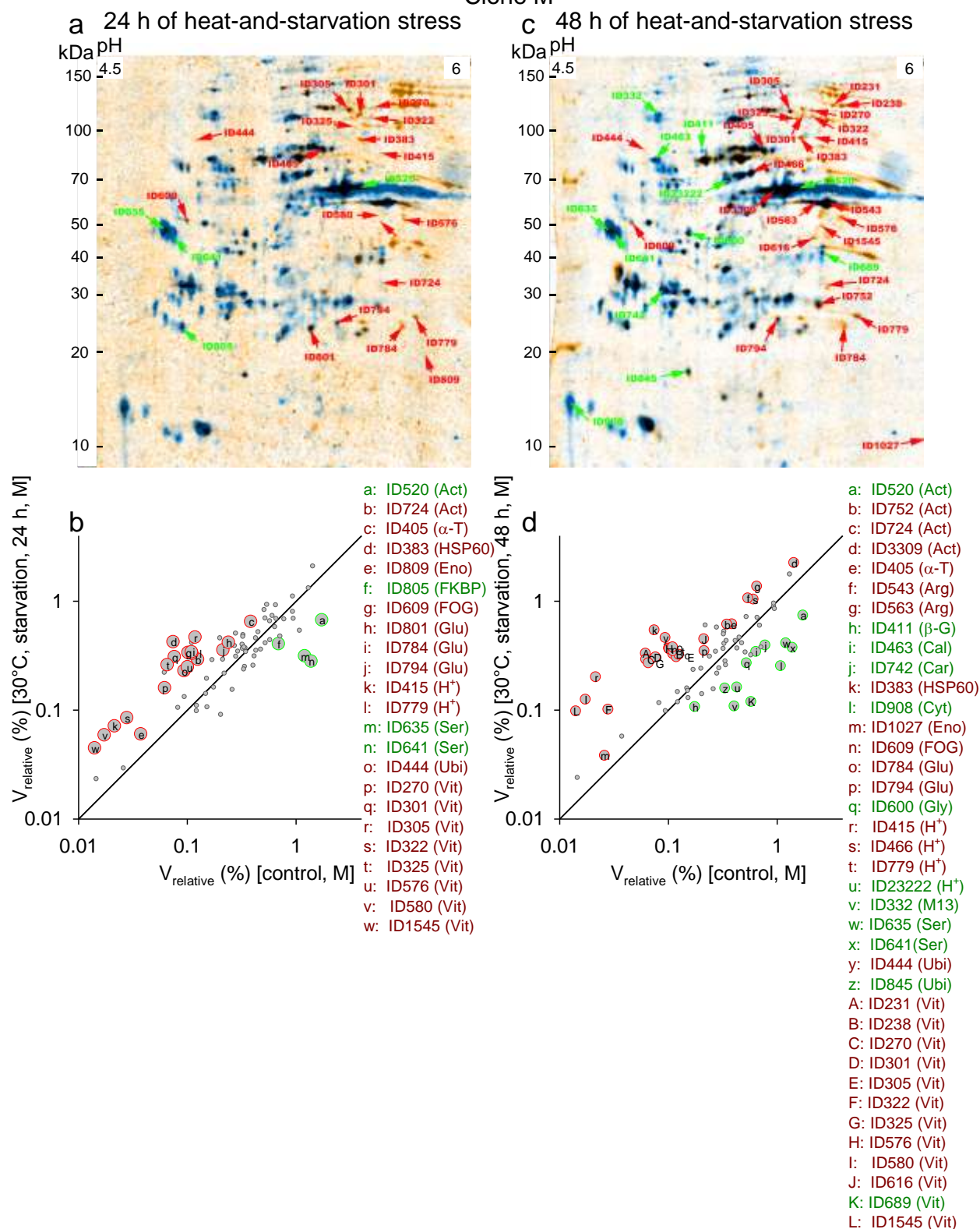

Supplement: Supplementary file 6 — Figure S5. Two-dimensional protein gels from the D. pulex clone M under heat-and-starvation stress. The 2D gels, which are fusion (averaged) images from a varying number (n) of gels (biological replicates, 25-30 animals each), show changes in protein expression in the D. pulex clone M after the acute exposure of control animals (20 °C, ad libitum feeding) (blue spots; n = 4) to (a) 24 h (orange spots; n = 5) or (c) 48 h (orange spots; n = 7) of heat-and-starvation stress (T = 30 °C, starvation). Red or green spot IDs mark significantly up- or downregulated proteins (t-tests, P < 0.05; see Table 5). The scatter plots show changes in expression level (Vrelative, relative spot volume) between control animals and animals exposed to heat-and-starvation stress (b, 24 h; d, 48 h) of significantly (large circles and letters) or non-significantly (small circles) up- or down-regulated proteins (data from a or c). Proteins, which were upregulated under heat-and-starvation stress, are found above the diagonal line. (PDF 194 kb) [file 12864_2018_4742_MOESM6_ESM.pdf]

Figure S6. Experimental design.

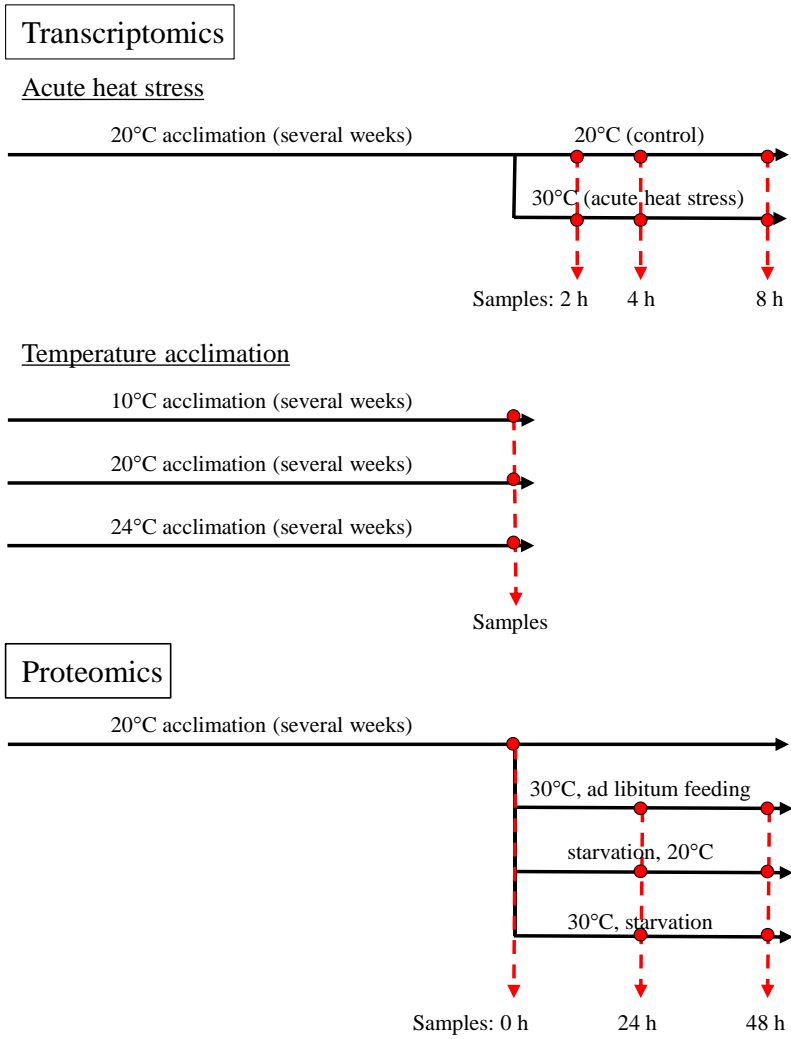

Supplement: Supplementary file 7 — Figure S6. Experimental design. (PDF 144 kb) [file 12864_2018_4742_MOESM7_ESM.pdf]
