# Supplementary material for: The transcriptomic and proteomic responses of Daphnia pulex to changes in temperature and food supply comprise environment-specific and clone-specific elements
Source: BMC Genomics. 2018 May 21;19:376. doi: 10.1186/s12864-018-4742-6 (PMC5963186; doi:10.1186/s12864-018-4742-6)
Supplement: Supplementary file 8 — Table S2. Labeling design of the microarrays. (PDF 83 kb) [file 12864_2018_4742_MOESM8_ESM.pdf]

Table S2. Labeling design of the microarrays.

| Array | Cy3 | Cy5 |
|---|---|---|
| 1 | 10°C (accl.) | 20°C (accl.) |
| 2 | 10°C (accl.) | 20°C (accl.) |
| 3 | 20°C (accl.) | 10°C (accl.) |
| 4 | 20°C (accl.) | 10°C (accl.) |
| 5 | 10°C (accl.) | 24°C (accl.) |
| 6 | 10°C (accl.) | 24°C (accl.) |
| 7 | 24°C (accl.) | 10°C (accl.) |
| 8 | 24°C (accl.) | 10°C (accl.) |
| 9 | 20°C (accl.) | 24°C (accl.) |
| 10 | 20°C (accl.) | 24°C (accl.) |
| 11 | 24°C (accl.) | 20°C (accl.) |
| 12 | 24°C (accl.) | 20°C (accl.) |
| 13 | 20°C (2 h) | 30°C (2 h) |
| 14 | 20°C (2 h) | 30°C (2 h) |
| 15 | 30°C (2 h) | 20°C (2 h) |
| 16 | 30°C (2 h) | 20°C (2 h) |
| 17 | 20°C (4 h) | 30°C (4 h) |
| 18 | 20°C (4 h) | 30°C (4 h) |
| 19 | 30°C (4 h) | 20°C (4 h) |
| 20 | 30°C (4 h) | 20°C (4 h) |
| 21 | 20°C (8 h) | 30°C (8 h) |
| 22 | 20°C (8 h) | 30°C (8 h) |
| 23 | 30°C (8 h) | 20°C (8 h) |
| 24 | 30°C (8 h) | 20°C (8 h) |

The table shows for each array the Cy3 or Cy5 labeling of samples. Abbreviations: 10°C, 20°C, or 24°C (accl.) means 10, 20, or 24°C acclimation; 20°C or 30°C (2 h, 4 h, or 8 h) means 2, 4, or 8 h under control (20°C) or stress conditions (30°C).
